# The Wisdom Researchers and the Elephant: An Integrative Model of Wise Behavior

**DOI:** 10.1177/10888683221094650

**Published:** 2022-06-02

**Authors:** Judith Glück, Nic M. Weststrate

**Affiliations:** 1 University of Klagenfurt, Austria; 2 University of Illinois Chicago, USA

**Keywords:** wisdom, wise behavior, wisdom measurement, wisdom development, wisdom trait, wisdom state

## Abstract

This article proposes an integrative model of wise behavior in real life. While current research findings depend considerably on how wisdom is conceptualized and measured, there are strong conceptual commonalities across psychological wisdom models. The proposed model integrates the components of several existing models into a dynamic framework explaining wise behavior. The article first specifies which real-life situations require wisdom and discusses characteristics of wise behavior. The core proposition of the model is that in challenging real-life situations, noncognitive wisdom components (an exploratory orientation, concern for others, and emotion regulation) moderate the effect of cognitive components (knowledge, metacognitive capacities, and self-reflection) on wise behavior. The model can explain the situation specificity of wisdom and the commonalities and differences between personal and general wisdom. Empirically, it accounts for the considerable variation in correlations among wisdom measures and between wisdom measures and other variables. The model has implications for the design of wisdom-fostering interventions and new wisdom measures.

Psychological wisdom research was, for many years, a small and not very visible field, but over the last few decades, it has been growing exponentially and attracting more and more attention both within and beyond psychology (Sternberg & Glück, 2019). One likely reason for this increasing interest is the current state of our world. Societies are more intelligent (Trahan et al., 2014) and educated (Hashiguchi et al., 2015) than ever before, but, despite many promising advancements in the medical and technological industries, we confront an unprecedented array of social and environmental problems, many of which are human-made. As we are faced with global challenges that require complex and balanced solutions, societies may be in urgent need of more wisdom, especially in our leaders (Grossmann & Brienza, 2018; Sternberg, 2018, 2019). Therefore, it seems worthwhile to find ways to foster wisdom, both through education and by creating structures that support the manifestation of wisdom in fields like politics, management, or the law. As any effort in this direction must be built on a solid foundation of theory and research, we propose an integrative model of wise behavior that unifies and extends important perspectives in the field.

## As Many Different Wisdom Theories as There Are Wisdom Researchers?

What do we know about wisdom at this point? More than 30 years of rigorous empirical inquiry have produced significant insights, and wisdom conceptions and methodological approaches have evolved considerably. However, one somewhat sobering discovery has been that study findings tend to depend on how wisdom is conceptualized and measured. For example, cross-sectional studies of the relationship between wisdom and age have found no association (Staudinger, 1999), a positive correlation (Grossmann et al., 2010), a negative correlation (Ardelt, 2003), an inverse U-shaped curve (Ardelt et al., 2018; Webster et al., 2014), and a U-shaped curve (Brienza et al., 2018). In addition, correlations between different measures of wisdom tend to be no larger than .30 (Glück et al., 2013; Taylor et al., 2011). At first sight, such findings suggest that different measures of wisdom are assessing quite different constructs. In addition, the lack of external criteria for determining who is wise makes it difficult to empirically decide which definition or measure of wisdom is “best.” Conceptually, however, there are clear commonalities across the different definitions. We have come to believe that the empirical inconsistencies largely arise from the fact that different conceptions of wisdom focus on different facets of a complex construct. Some researchers have compared the current state of wisdom psychology to the proverbial “blind men and the elephant” (Grossmann, Weststrate, Ardelt, et al., 2020; Grossmann, Weststrate, Ferrari, & Brienza, 2020; Sternberg et al., in press). In this ancient story from India, a group of blind men learn what an elephant is by touching it. Because of the elephant’s size, each man can only touch one part, and they build their ideas of the elephant based on their respective parts. As a result, their descriptions of the elephant are very different. In other words, people tend to define complex concepts based on the parts they are most familiar with. In this way, researchers with different backgrounds have focused on different aspects of the complex construct of wisdom. Current wisdom models tend to focus either on cognitive components, such as rich self- and life-knowledge or metacognition, or on personality components, such as compassion or openness. The model proposed here is the first to integrate these two broad domains—the “head” and the “heart” of the elephant—by arguing that *both* are required for acting wisely in real life. In a nutshell, the model proposes that in real life, wisdom manifests itself in situations that are important, difficult, uncertain, and emotionally challenging. Neither cognitive nor personality components of wisdom alone are sufficient to understand real-life wisdom (Glück, 2020a). Individuals who show high levels of wise reasoning in psychologists’ labs may not act equally wisely in difficult real-life situations unless they remain calm, empathetic, and open-minded even under high levels of stress. On the other hand, individuals who are calm, empathetic, and open-minded but do not have wisdom-related knowledge and reasoning skills will not be able to act wisely in real life either.

By integrating the two approaches, our model can answer several open questions in the field: why wisdom varies across situations, whether personal and general wisdom are separate constructs, why the correlations between different wisdom models are often low, and why relationships between wisdom and other constructs are so inconsistent. The model also has implications for how wisdom can be fostered and how measures of wisdom could be optimized. In the following, we first discuss the characteristics of those real-life situations that most require wisdom and what we know about wise behavior in such situations. Then, we briefly review psychological models and measures of wisdom, focusing on their relevance for dealing with real-life situations. Next, we introduce the new integrative wisdom model. Finally, we discuss what the integrative model contributes to current debates and point out its limitations and important open questions.

## When and Where Do We Need Wisdom, and How Does It Manifest Itself?

### Characteristics of Wisdom-Requiring Situations

Before we discuss the characteristics of wise behavior, we first need to specify the situations in which wise behavior manifests itself most clearly. While highly wise individuals probably live more wisely than most of us in many ways, including a focus on eudaimonic rather than hedonic well-being and universalistic and self-directed value orientations (Bauer et al., 2019; Glück et al., 2020; Weststrate & Glück, 2017b), wisdom arguably manifests itself most clearly in the face of life challenges. Research on wisdom nominations and autobiographical wisdom suggests that people typically associate wise behavior with difficult, complex, and uncertain life situations (e.g., Glück et al., 2005; Montgomery et al., 2002; Yang, 2008). For example, Glück et al. (2005) and Yang (2008) interviewed participants about situations where they had been wise. Most participants reported situations where they either faced or supported someone else facing (a) a difficult life decision or moral dilemma, (b) a negative event or conflict, or (c) a challenging long-term situation. Wisdom researchers seem to share this association of wise behavior with life challenges given that performance measures of wisdom typically present participants with difficult life problems (e.g., Baltes & Smith, 1990; Baltes & Staudinger, 2000; Grossmann et al., 2010, 2013; Grossmann & Kross, 2014; Kross & Grossmann, 2012; Staudinger et al., 1994).

### Characteristics of Wise Behavior in Challenging Situations

How do wise individuals deal with life problems? Surprisingly little empirical research has looked at the relationships between wisdom and the way people deal with difficult situations in real life. Most evidence comes from research on folk conceptions of wisdom, as several studies asked participants to rate lists of characteristics for their relevance to wisdom, and those lists often included concrete behaviors (Weststrate et al., 2019). In addition, three qualitative and mixed-methods studies specifically analyzed people’s narratives of situations in which they thought that they or someone else had acted wisely (Glück et al., 2005; Montgomery et al., 2002; Yang, 2008). Although what people report as wise may not always actually be wise, these studies offer some interesting insights into real-life perceptions of wisdom. Across the three studies, most wise behaviors achieved one or more of three broad outcomes:

1. *Resolving difficult short-term or long-term problems*. In all three studies, wise many participants felt that wise behavior had resolved difficult short-term or longer-term situations. For example, people used wisdom to resolve family conflicts, to cope with a serious illness of themselves or a family member, or to make difficult life decisions (Glück et al., 2005). Wisdom was also used to provide guidance and advice to family members or friends in challenging situations or to solve complex problems faced by larger institutions (Glück et al., 2005; Montgomery et al., 2002; Yang, 2008).
2. *Supporting others or contributing to a larger common good.* Many participants talked about wise behavior achieving a common good. This ranged from providing guidance to family or friends to resolving work-related or institutional conflicts, achieving positive change in larger communities or institutions, and even, in Yang’s (2008) study, having a positive impact on a whole nation. Montgomery et al. (2002) argued that offering helpful advice or guidance was the main form that wise behavior took.
3. *Knowing and doing what is right.* In all three studies, some participants related wisdom to knowing what is right for oneself (or another person to whom one provides guidance) and making life decisions or finding ways to live (or enable the other person to live) according to these insights. For some participants, this involved doing what was morally right even in the face of negative personal consequences (Glück et al., 2005; Yang, 2008). Consistent with the philosophical notion that wisdom is about living a “good life” (Grimm, 2015; Kekes, 1983; Ryan, 1999), these participants saw the wisdom in pursuing one’s individual path to a rewarding and meaningful life, even against resistance from others or strict societal norms.

But how do wise individuals actually achieve those outcomes? In the following, we draw on research on folk conceptions of wisdom and empirical wisdom research that identified characteristics of wise behavior in the face of difficult life problems. We structure this review sequentially into (a) gaining an unbiased understanding of the problem situation, (b) thinking about the problem and (pathways toward) possible solutions, and (c) taking steps toward implementing the best possible solution. Table 1 gives an overview of the behaviors identified as characteristic of wisdom in folk-conception studies.

1. *Gaining an unbiased understanding of the problem situation.* First, wise behavior involves collecting as much knowledge as possible to understand the factual but also the emotional and social aspects of a complex problem situation. Wise individuals “take a step back” mentally to gain an objective picture of both the larger context and the background of the problem and its in-depth details. This typically involves listening to the perspectives of the people or groups involved (Sternberg, 1998, 2019) in a calm, open-minded, respectful, and empathetic way (Itzchakov et al., 2018).
2. *Thinking about the problem and (pathways toward) possible solutions, guided by ethical principles.* Once wise individuals have a clear picture of the problem in its complexity, they draw on their wisdom-related knowledge and expertise to consider ways to balance the various interests involved and work toward possible solutions that maximize a common good. This often involves consulting others for advice (Igarashi et al., 2018). The more they are personally and emotionally involved, the more will wise individuals reflect on their own biases and regulate their own emotions. With complex problems, the first goal may not be to identify a solution, but to come up with workable pathways toward a solution, taking both short-term and long-term consequences into account (Sternberg, 2019).
3. *Proposing and/or implementing the best possible solution.* Once a relatively best pathway toward a solution has been identified, wise individuals will try to ensure that it is pursued. Obviously, with complex and uncertain problems, a single best solution rarely exists, so a stepwise, iterative process of trying out, evaluating, and refining will often be necessary. Wise individuals will not typically tell people what to do. They will use their experience, morality, sincerity, and social skills to provide guidance and support (Montgomery et al., 2002).

Should a person’s behavior be considered wise if it does not lead to a positive outcome? In studies of autobiographical wisdom memories, participants typically tell stories of difficult situations that ended well; in fact, a positive outcome seems to be viewed as necessary for considering one’s own behavior as wise (Bluck & Glück, 2004; Glück et al., 2005). In contrast, we suggest that behavior should be considered wise if it shows the characteristics described earlier, regardless of whether it leads to a positive outcome, just as aggressive behavior would still be considered aggressive if it caused no harm at all.^1^ Wise behavior is most likely to achieve a positive outcome in highly complex and difficult life situations, but some such situations are just impossible to resolve positively. Ideally, wise behavior would maximize the probability of a positive outcome in a given situation, but that probability would still be less than 100%.

**Table 1.** Characteristics of Wise Behavior According to Studies of Folk Conceptions of Wisdom.

(1) Gaining an unbiased understanding of the problem	(2) Thinking about the problem and possible solutions	(3) Suggesting/implementing solutions:
• A good listener (Holliday & Chandler, 1986; König & Glück, 2013; Sternberg, 1985) • Ability to understand complex issues (Glück & Bluck, 2011; König & Glück, 2013) • Able to see through things (Sternberg, 1985) • Aware (Holliday & Chandler, 1986) • Being critical (König & Glück, 2013) • Considering others’ situation/life context (Glück et al., 2005) • Detachment (Kałużna-Wielobób, 2014) • Listens to all sides of an issue (Sternberg, 1985) • Makes connections and distinctions (Sternberg, 1985) • Objectivity (Kałużna-Wielobób, 2014) • Observant/perceptive (Clayton & Birren, 1980; Holliday & Chandler, 1986; Sternberg, 1985) • Seeing the whole (König & Glück, 2013) • Seeks out information (Sternberg, 1985) • Sees and considers all points of view (Holliday & Chandler, 1986) • Sees the essence of situations (Holliday & Chandler, 1986) • Sees things within larger context (Holliday & Chandler, 1986) • Takes in a complex situation at a glance (Yang, 2001) • Taking others’ perspectives, accepting different values (Glück et al., 2005) • Understands people (Hershey & Farrell, 1997; Sternberg, 1985)	• Ability to apply knowledge (Sternberg, 1985) • Able to flexibly/creatively apply knowledge to daily life (Yang, 2001) • Considers all options in a situation (Holliday & Chandler, 1986) • Dealing with one’s own emotions (Glück et al., 2005) • Problem solving (Chen et al., 2014) • Problem-solving ability (Holliday & Chandler, 1986; Sternberg, 1985) • Recognition and management of uncertainty (Glück et al., 2005) • Reliance on factual or procedural knowledge (Glück et al., 2005) • Taking others’ advice (Glück et al., 2005) • Thinking things through carefully (Glück et al., 2005) • Thinks beyond what the ordinary person thinks (Yang, 2001) • Thinks clearly (Yang, 2001) • Trusting oneself and one’s intuition (Glück et al., 2005)	• Action strategies (Chen et al., 2014) • Being honest and responsible (Glück et al., 2005) • Being willing to take a risk (Glück et al., 2005) • Being willing to take time with things (Glück et al., 2005) • Communication skills (Kałużna-Wielobób, 2014) • Drawing on compassion in providing guidance (Montgomery et al., 2002) • Drawing on knowledge and experience in providing guidance (Montgomery et al., 2002) • Drawing on moral principles in providing guidance (Montgomery et al., 2002) • Knows when to give and not give advice (Holliday & Chandler, 1986) • Making compromises (Glück et al., 2005) • Offers alternative solutions to problems (Brezina, 2010) • Offers solutions on the right side of truth (Sternberg, 1985) • Practical use of knowledge/skills (Kałużna-Wielobób, 2014) • Pragmatic (Clayton & Birren, 1980) • Providing problem-focused or emotion-focused support (Glück et al., 2005) • Seeks compromise (Brezina, 2010) • Sincere (Hershey & Farrell, 1997) • Sincere and warm-hearted (Yang, 2001) • Skilled in everyday affairs (Holliday & Chandler, 1986) • Socially competent (König & Glück, 2013) • Standing by one’s values or goals (Glück et al., 2005) • Taking control of situations (Glück et al., 2005)

By characterizing wise behavior, we have set the stage for a review of psychological models of wisdom. Which underlying psychological qualities and capacities enable people to act wisely in challenging situations?

## Psychological Definitions of Wisdom

Except for a few early theoretical accounts (Erikson, 1959; Hall, 1922), psychological wisdom research began in earnest in the late 1970s and early 1980s. Several researchers at the time considered it wise to start by looking at the wisdom conceptions of people outside academia (e.g., Clayton & Birren, 1980; Holliday & Chandler, 1986; Sternberg, 1985; see Tables 1 and 3); this field has grown considerably over time (overview in Weststrate et al., 2019). In some cases, these informal theories provided the foundation for psychologists’ formal definitions of wisdom (e.g., Ardelt, 2003; Sternberg, 1998; Yang, 2001), on which we focus in the following.

We review those definitions of wisdom that have strong theoretical underpinnings, established measurement models, and produced substantial amounts of empirical research. Table 2 summarizes those definitions and the measures of wisdom corresponding to them. Other researchers have proposed additional definitions (e.g., Brown, 2004; Brown & Greene, 2006; Brugman, 2006; Jason et al., 2004, 2001; Knight & Laidlaw, 2009; McKee & Barber, 1999; Moraitou & Efklides, 2012; Sternberg, 1998, 2019; M. L. Thomas et al., 2019; Yang, 2001); for additional theoretical reviews see Bangen et al., 2013; Grossmann, Weststrate, Ardelt, et al., 2020; Karami et al., 2020; Sternberg & Karami, 2021; Walsh, 2015).

**Table 2.** Overview of Models and Measures of Wisdom.

Model and authors	Definition	Dimensions/criteria	Measure/problems/items
**Cognitive-Focused Models:**			
**Berlin Wisdom Model** (Baltes & Staudinger, 2000)	Wisdom-related knowledge: Expert knowledge about the fundamental pragmatics of life	(1) Factual knowledge about the issue (2) Procedural knowledge about the issue (3) Lifespan contextualism (4) Value relativism (5) Recognition and management of uncertainty	**Berlin Wisdom Paradigm (BWP):** Thinking aloud about brief descriptions of difficult life problems, e.g., “A 15-year-old girl wants to move out of her family home immediately. What could one consider and to in such a situation?” (Staudinger et al., 1994)
**BWP correlates:** openness to experience, personal growth, intelligence, life experience, emotional competence, creativity, cognitive styles, affective involvement, growth-related and other-enhancing values (Baltes & Staudinger, 2000; Glück et al., 2013; Kunzmann & Baltes, 2003; Staudinger et al., 1997, 1998)			
**Bremen Wisdom Model** (Mickler & Staudinger, 2008)	Self-related wisdom: sound judgment and deep insight with regard to difficult and uncertain matters of one’ own life	(1) Rich self-knowledge (2) Heuristics of growth and self-regulation (3) Interrelating the self (4) Self-relativism (5) Tolerance of ambiguity	**Bremen Wisdom Paradigm (BrWP):** Interview about the participant as a friend, both generally and in difficult situations (Mickler & Staudinger, 2008)
**BrWP correlates:** intelligence, openness to experience, maturity, life experience, age (Mickler & Staudinger, 2008)			
**Wise-reasoning Model** (e.g., Grossmann, 2017)	Wise reasoning: “the use of certain types of pragmatic reasoning to navigate important challenges of social life” (Grossmann et al., 2010, p. 7246)	(1) Intellectual humility (2) Seeing others’ perspectives (2) Integrating different perspectives (4) Recognizing uncertainty and change	**Wise-reasoning Paradigm (WRP):** Vignettes about problems concerning personal or large-scale societal issues; participants are asked how the situation might unfold and why; responses written or spoken (e.g., Grossmann et al., 2010) **Situated Wise Reasoning Scale (SWIS):** 21 items referring to a recent conflict, e.g.: “Put myself in the other person’s shoes.” “Double-checked whether the other person's opinions might be correct.” (Brienza et al., 2018)
**WRP correlates:** crystallized intelligence, agreeableness, aspects of well-being (Grossmann et al., 2013) **SWIS correlates:** Openness, extraversion, intellect, emotional intelligence, emotion regulation, mindfulness, reflection (Brienza et al., 2018)			
**Personality-Focused Models:**			
**Self-Transcendence Model** (Aldwin et al., 2019; Levenson et al., 2005	Self-transcendence: independence from external self-definitions and the dissolution of rigid boundaries between the self and others	Four developmental stages (1) Self-knowledge (2) Non-attachment (3) Integration (4) Self-transcendence	**Adult Self-Transcendence Inventory (ASTI):** 35-items, e.g., “I can learn a lot from others.” “My peace of mind is not easily upset.” “I feel that my individual life is part of a greater whole.” (Koller et al., 2017; Levenson, et al., 2005)
**ASTI correlates:** openness to experience, personal growth, self-acceptance, emotional competence, extraversion, empathy, meditation practice, egalitarianism (Glück et al., 2013; Le & Levenson, 2005; Levenson et al., 2005			
**Three-Dimensional Wisdom Model** (Ardelt, 2003)	Wisdom as a combination of personality traits that enable individuals to take others’ perspectives and overcome biases and blind spots, learn from life, and care for others	(1) Cognitive dimension (2) Reflective dimension (3) Compassionate dimension	**Three-Dimensional Wisdom Scale (3D-WS)** 39 items, e.g.: “Things often go wrong for me by no fault of my own” (reverse-coded “Sometimes I feel a real compassion for everyone” “Ignorance is bliss” (reverse-coded) (Ardelt, 2003)
**3D-WS correlates:** Openness to experience, personal growth, emotional competence, empathy, mastery, purpose in life, forgiveness, well-being (Ardelt, 2003, 2011; Glück et al., 2013)			
**Developmental Models:**			
**HERO(E) Model of Wisdom** (Webster, 2003, 2007)	“the competence in, intention to, and application of, critical life experiences to facilitate the optimal development of self and others” (Webster, 2007, p.164)	(1) Critical life experience (2) Openness (3) Emotional regulation (4) Reminiscence and reflectiveness (5) Humor	**Self-Assessed Wisdom Scale (SAWS):** 40 items, e.g.: “I have had to make many important life decisions.” “I can regulate my emotions when the situation calls for it.” “I can chuckle at personal embarrassments.” (Webster, 2007)
**SAWS correlates:** Openness to experience, personal growth, emotional competence, self-efficacy, ego integrity, forgiveness, personal well-being, empathy, generativity, and positive psychosocial values (Glück et al., 2013; Taylor et al., 2011; Webster, 2003, 2007, 2010)			
**MORE Life Experience Model** (Glück & Bluck, 2013; Glück et al., 2019)	Psychological resources that foster growth in wisdom as individuals reflect on life challenges	(1) Managing uncertainty and uncontrollability (2) Openness to new perspectives and experiences (3) Reflectivity (4) Emotional sensitivity and emotion regulation	**MORE Life Experience Interview (MORE):** Interview about difficult autobiographical events or conflicts (Glück et al., 2019)
**MORE Correlates:** Crystallized intelligence, openness to experience, interpersonal emotional competence, self-reflection, self-direction (Glück et al., 2020)			

*Note.* BWP = Berlin Wisdom Paradigm; WRP = Wise-reasoning Paradigm; SWIS = Situated Wise Reasoning Scale; ASTI = Adult Self-Transcendence Inventory; 3D-WS = Three-Dimensional Wisdom Scale; SAWS = Self-Assessed Wisdom Scale.

### Cognitive-Focused Models of Wisdom

Early psychological models of wisdom focused on cognitive aspects of wisdom, drawing on lines of research such as neo-Piagetian conceptions of postformal cognition (Kramer, 1990; Labouvie-Vief, 1990), expertise theory (Baltes & Smith, 1990), and practical intelligence (Sternberg, 1998). The first comprehensive wisdom-research program was based on the so-called Berlin Wisdom Model (Baltes & Smith, 1990; Baltes & Staudinger, 2000), which defines wisdom as expert knowledge about the important and difficult issues of human life. According to the Berlin model, wise thinking about difficult life problems is characterized by high levels of factual and procedural life knowledge and by an awareness of the differences in values and life contexts that shape people’s perspectives and of the uncertainty and unpredictability of life (Baltes & Staudinger, 2000).

Building on the Berlin model, Staudinger et al. (2005; see also Mickler & Staudinger, 2008; Staudinger, 2019) proposed to distinguish between general wisdom (wisdom about life and people) and personal wisdom (wisdom about oneself and one’s own life). Personal wisdom manifests itself in how people deal with challenges in their own life, which may be quite different from how they think about challenges in the lives of other people. The Bremen Wisdom Paradigm (Mickler & Staudinger, 2008) includes five criteria for personal wisdom that run parallel to the five criteria of the Berlin Wisdom Paradigm but refer to how people think about themselves and their own life problems.

The research program of Igor Grossmann and colleagues also builds on the Berlin model and on research on dialectical thinking. It focuses on wise reasoning, which is characterized by metacognitive processes involving intellectual humility, awareness of uncertainty, and consideration of different contexts and perspectives (for overviews see, e.g., Grossmann, 2017; Grossmann, Weststrate, Ardelt, et al., 2020; Oakes et al., 2019). Importantly, Grossmann and colleagues have demonstrated that wise reasoning varies considerably between situations. Americans reason more wisely, for example, when they think about U.S. politics from an Icelander’s perspective than from their own (Kross & Grossmann, 2012), when they imagine a problem concerning someone else than concerning themselves (Grossmann & Kross, 2014), or when they are in a more positive mood and better able to regulate their emotions (Grossmann et al., 2016). Accordingly, wise reasoning can be fostered by instructing people to de-center their perspective.

Sternberg’s balance theory of wisdom (Sternberg, 1998, 2019) focuses less on how individuals think about life problems and more on the problem solutions they come up with. According to Sternberg, wise solutions balance (a) the different intrapersonal, interpersonal, and extrapersonal interests involved with the aim of achieving a common good, (b) possible ways to respond to a challenging situation (adapting to the environment, changing the environment, or leaving the environment altogether), and (c) the short-term and long-term consequences of any course of action.

To summarize, cognitive-focused models of wisdom describe how wise people think about life problems. Wise thinking is characterized by an awareness of the multiperspectival nature of complex situations, the limitations of one’s own knowledge, and the unpredictability of the future. Wise thinking is assumed to produce problem solutions that are balanced across the different needs involved and that take long-term as well as short-term outcomes into account. Importantly, the extent to which people are able to reason wisely is influenced by situational characteristics. People reason more wisely when they are willing and able to consider other perspectives than their own.

### Personality-Focused Models of Wisdom

Personality-focused models entered the field around 2000. Partly building on criticisms of the cognitive focus of existing conceptions of wisdom (Ardelt, 2004), they emphasize personality-related, emotional, and motivational components of wisdom. Most prominently, the Three-Dimensional Wisdom Model (Ardelt, 2003) defines wisdom as a combination of three personality dimensions. The reflective dimension is a willingness to look at things from different perspectives to gain a broader, less subjective understanding. The cognitive dimension is “a desire to know the truth, i.e., to comprehend the significance and deeper meaning of phenomena and events, particularly with regard to intrapersonal and interpersonal matters” (Ardelt, 2004, p. 275). The affective (or, in more recent publications, compassionate) dimension is defined as sympathetic and compassionate love for others. Thus, Ardelt’s model is quite consistent with cognitive-focused wisdom models, as it includes personality dimensions that motivate people to gain wisdom-related knowledge and consider different perspectives on complex problems. However, it adds compassion as an important factor that may motivate people to support others and seek a common good in difficult situations.

Moving further away from cognitive aspects of wisdom, Michael R. Levenson, Carolyn Aldwin, and colleagues (Aldwin et al., 2019; Igarashi et al., 2018; Levenson et al., 2005) argue that the core quality of wisdom is self-transcendence: feeling connected to people, other generations, and nature; having a sense of meaning and purpose; and experiencing positive emotions such as joy, inner peace, and awe (Aldwin et al., 2019). “Thus, [self-transcendence] is the antithesis of the narcissism and materialistic strivings which are so often at the heart of psychological distress” (Aldwin et al., 2019, p. 137). This conception of wisdom is clearly more distant from cognitive-focused models, but it shares with them the orientation toward a common good. Self-transcendent individuals would be at peace with themselves and care about the needs of others even in highly challenging situations.

### Developmental Models of Wisdom

A third group of wisdom models focuses on wisdom as an outcome of learning from life through the reflection of previous experiences. These models assume that both cognitive components, such as self-reflection, and non-cognitive components, such as openness, are necessary for gaining wise insights from life experiences. Jeffrey Dean Webster’s HERO(E) model (Webster, 2003, 2007) defines wisdom as the willingness and ability to apply insights gained from life experiences to facilitate the optimal development of oneself and others (Webster, 2007). Critical life experiences are, therefore, viewed as a precondition for the development of wisdom. Openness and a willingness to reminisce and reflect are necessary for learning from such experiences, and emotion regulation and humor help wise individuals to deal with difficult experiences and make sense of them.

Glück and Bluck (2013; Glück et al., 2019) proposed the MORE Life Experience Model as a theory of how wisdom develops and how it manifests itself in difficult situations. They consider life-changing events as the main catalysts of the development of wisdom. To grow wiser from such experiences, however, certain psychological resources are necessary. According to Glück et al. (2019), these resources are the ability to manage uncertainty and uncontrollability, openness to divergent perspectives and new experiences; reflectivity (see Weststrate & Glück, 2017a); and emotional sensitivity and regulation.

To summarize, at first sight, existing definitions of wisdom vary considerably. They range from wisdom as expert knowledge to wisdom as interconnectedness, from wisdom as personality to wisdom as learning from life. The metaphor of the “blind men and the elephant” seems to fit wisdom research quite well. Wisdom researchers certainly differ from the blind men in the proverb in that they are perfectly aware (and generally respectful) of each other’s conceptions, but different labs have each built their research on their own model and measures. To understand how wisdom operates in real life, however, many different facets from different models are relevant. As we will argue in the following, in those challenging situations that most require wisdom, the noncognitive components of wisdom—an exploratory orientation, concern for others, and emotion regulation—are necessary for full utilization of the cognitive components—knowledge, metacognitive capacities, and self-reflection. First, however, we discuss how the differences between conceptions are mirrored in differences between measures of wisdom.

## Measuring Wisdom

Researchers have long grappled with the challenge of how to best measure wisdom. Typically, wisdom is assessed either using open-ended performance measures or self-report scales (Glück, 2018; Glück et al., 2013; Kunzmann, 2019; Webster, 2019). We briefly describe each approach in turn; more detailed information on the various measures was presented in Table 2.

### Performance Measures

In performance measures such as the Berlin Wisdom Paradigm or the Wise Reasoning Paradigm, participants respond in speaking or writing to wisdom-requiring problems. Responses are scored by trained raters according to specific wisdom criteria. Accordingly, performance-based wisdom measures, especially those administered verbally, require a high amount of effort for administration, transcription, and scoring (Glück, 2018; Kunzmann, 2019). In addition, it is not yet clear to what extent performance measures are valid indicators of how a person would act in the face of a difficult real-life situation. As Grossmann and Kross (2014) have shown, for example, participants reason more wisely about the same problem if they imagine it concerning someone else than if they imagine it concerning themselves. The emotional arousal and increased self-focus that real-life challenges often involve may limit the extent to which people can utilize their wisdom-related knowledge and reasoning strategies.

### Self-Report Measures

The most popular self-report measures of wisdom are the Three-Dimensional Wisdom Scale (3D-WS; Ardelt, 2003), the Self-Assessed Wisdom Scale (SAWS; Webster, 2003, 2007), and the Adult Self-Transcendence Inventory (ASTI; Levenson et al., 2005); sample items were presented in Table 2. Self-report scales are easy to administer and score, but they have an inherent problem when it comes to measuring wisdom: If wise individuals are more self-reflective than other people, they might be more aware of their own weaknesses and blind spots and might therefore describe themselves less favorably in self-report scales than less wise individuals do (Aldwin, 2009; Glück, 2018; see also the “modesty paradox” discussed by Tangney, 2009). In addition, as Brienza et al. (2018) pointed out, classical self-report scales assess *typical* behavior; they do not focus on how participants act in those rare, challenging situations that most require wisdom. Thus, again, their validity as indicators of how a person would deal with a real-life challenge may be limited.

Recently, several research groups have attempted to design measures that come closer to real life in terms of emotional immersion. For example, S. Thomas and Kunzmann (2014) used videos of real couples discussing relationship problems. Glück and colleagues interviewed participants about autobiographical life challenges (Glück et al., 2019; König & Glück, 2014; Weststrate & Glück, 2017a). Brienza and colleagues (2018) developed the Situated Wise Reasoning Scale, a self-report measure of wise reasoning in a recent real-life conflict. Clearly, wisdom measurement is making progress toward capturing the emotional aspects inherent to real-life problems (Glück, 2018). We will discuss the implications of the integrative wisdom model for measurement later in this article.

### Relationships Across Measures

Empirical relationships between different measures of wisdom tend to be relatively weak. Measures assessing the same construct ought to be highly correlated, but correlations between wisdom measures seldom exceed .30 (Glück et al., 2013; Taylor et al., 2011). This puzzle is not fully explained by method variance: Correlations among self-report scales or among performance measures are not necessarily higher than correlations across the two approaches. For example, Mickler and Staudinger (2008) found a correlation of .27 between the Berlin Wisdom Paradigm and the Bremen Wisdom Paradigm; Glück et al. (2013) found a correlation of .26 between the Three-Dimensional Wisdom Scale and the Self-Assessed Wisdom Scale.

These low correlations imply that study results may be quite dependent on the specific instruments used. As discussed earlier, the relationship of wisdom with age varies considerably by measure (Glück, 2019a), and so does the relationship of wisdom with other variables such as well-being, empathy, or intelligence (Glück, 2015; Glück et al., 2013). These findings bring up the question whether “wisdom” as an overarching psychological quality even exists. If the correlations between different wisdom measures are no higher than the correlations of wisdom measures with other variables (Glück et al., 2013), one might conclude that the different wisdom measures simply assess different constructs. That would be a significant problem for a cumulative science of wisdom. After many years at the frontlines of wisdom research, however, we have come to believe in the “elephant theory”: that the different measures of wisdom are compatible and complementary rather than contradictory, focusing on different facets of one complex phenomenon and operationalizing them in different ways, and that all these facets come together in real-life manifestations of wisdom.

We stopped looking for the “best” or “correct” definition of wisdom when we analyzed autobiographical narratives of life challenges. Participants completed four wisdom measures and were interviewed about two autobiographical experiences: a serious conflict and an unspecified difficult event. Interview transcripts were scored by independent raters with respect to criteria derived from four different wisdom conceptions: the Berlin Wisdom Paradigm (Baltes & Staudinger, 2000), the Three-Dimensional Wisdom Model (Ardelt, 2003), the Bremen Wisdom Paradigm (Mickler & Staudinger, 2008), and the MORE Life Experience Model (Glück & Bluck, 2013). In addition, a group of untrained students rated the transcripts according to their own subjective conceptions of wisdom. We found two interesting results. First, the correlations between the four original measures of wisdom were mostly below .30. Second, however, the correlations between the interview-transcript ratings for the four different wisdom models were in the .70 to .80 range, and the ratings for all four wisdom conceptions had correlations above .60 with the students’ subjective wisdom ratings. In other words, a person might quite easily have a high wisdom score on the 3D-WS and a low wisdom score in the Berlin Wisdom Paradigm or vice versa (*r* = .25). But if an interview transcript about an autobiographical life challenge was rated as high on the dimensions of the 3D-WS, that transcript was probably also rated as high on the criteria of the Berlin Wisdom Paradigm (*r* = .72). These findings suggest that the low correlations between measures of wisdom may mostly be caused by differences in the *measures*, which may have distracted us from seeing that our *conceptions* of wisdom are not incompatible after all. When people were talking about dealing with difficult challenges from their own lives, different components of wisdom seemed to be much more closely related than when wisdom was measured in more abstract terms.

These findings got us thinking about how the various definitions can be integrated into one unifying model of wise behavior. Our model proposes that neither the cognitive nor the non-cognitive characteristics of wisdom alone are sufficient for acting wisely in real life, such as dealing with highly distressed individuals, resolving entrenched family conflicts, or making balanced, sustainable political decisions in the face of urgent crises. Wisdom-related cognitive capacities, which enable an individual to respond wisely to theoretical wisdom problems in psychologists’ labs (Glück, 2020a), are necessary but not sufficient for wise behavior in highly challenging real-life situations. In such cases, an exploratory orientation, concern for others, and emotion regulation are necessary for acting wisely as well. At the same time, these noncognitive qualities will not lead to wise behavior unless they are combined with high levels of wisdom-related knowledge, metacognitive capacities, and self-reflection. Thus, we propose that in difficult real-world situations, both the cognitive and the non-cognitive components are necessary to produce wise behavior. Moreover, we believe that a stronger focus on wise behavior is important for bringing wisdom research closer to the real world given that both cognition and personality can be unreliable predictors of actual behavior (e.g., Hassan et al., 2016; Sheeran & Webb, 2016).

This article presents the “elephant model” of wise behavior and demonstrates how it can explain inconsistent findings and resolve current controversies in wisdom research. Specifically, we believe that our model can (a) resolve the tension between conceptions of wisdom as a stable trait and wisdom as a situation-dependent state; (b) account for the differences between general and personal wisdom, that is, between people’s wisdom about life in general and their wisdom about themselves and their own life; (c) explain the low correlations between different measures of wisdom; and (d) explain the inconsistent correlations of wisdom measures with other variables. In addition, the model has implications for the development of effective short-term and long-term wisdom interventions and valid wisdom measures.

## Developing an Integrative Model of Wise Behavior

Before describing the model, we explain how potential components were evaluated for inclusion in the model.

### Distinguishing Components of Wisdom From Other Related Variables

Although the focus of the new model is on integrating existing models of wisdom, not all components of all models were included. How should one decide whether a psychological construct is an actual component or just a correlate of wisdom? Many constructs are not components of wisdom but still conceptually and empirically related to it. For instance, there are qualities that we would call *threshold variables*—characteristics of which a person needs a certain level to be wise. A certain amount of intelligence, for example, is probably necessary for individuals to act wisely in difficult situations and to become wiser over time (Glück, 2020b; Glück & Scherpf, in press; Webster, 2010). Beyond that threshold, however, increases in intelligence are unlikely to result in increases in wisdom. Also, there are *outcome variables*—variables that result from a person’s wisdom but are not a constituent part of it, such as life satisfaction (Ardelt, 2016; Glück et al., in press; Weststrate & Glück, 2017b), gratitude (König & Glück, 2014), or forgiveness (Taylor et al., 2011). In addition, the importance of certain qualities for wisdom may change over the lifespan. For example, openness to experience, empathy, or intelligence may be early-life precursors—*antecedent variables*—that help some individuals gain wisdom from experiences but become less important as life knowledge and self-regulatory capacities grow (Glück et al., 2019; Pasupathi et al., 2001).

In addition, it is difficult to evaluate how well a model of wisdom really describes wisdom. In other domains of expertise involving complex, ill-defined real-world problems, a “good” decision or outcome can be clearly defined—in firefighting, for example, we can say that expert performance is one in which the fire is brought under control in a safe and efficient manner. From there, we can work backward to identify the components of expert performance (Swartwood, 2013). The outcome of wisdom is more challenging to pin down, as we do not have clear external criteria for wise persons or wise behavior. Elsewhere (Glück, 2020a), we have suggested four possible criteria for the validity of a wisdom model which we discuss briefly in the following.

### Criteria for Including Components in the Models

#### Consistency with nonexperts’ conceptions of wisdom

First, as wisdom is highly relevant to human lives and people are able to define it with relative ease (Weststrate et al., 2019), the model should be consistent with nonexpert views of wisdom. This heuristic may be less applicable to constructs that are less well represented in the linguistic repertoire of people outside academia or that require in-depth technical knowledge, but wisdom is a concept that even children are familiar with (Asadi et al., 2019; Glück et al., 2012). All components of the integrative model are consistent with research on (at least Western) people’s views of wisdom (Weststrate et al., 2019); the respective evidence is presented in the first column of Table 3.

**Table 3.** Evidence for Including Each Component Into the Integrative Wisdom Model.

Component	Related characteristics in folk conceptions	Wisdom models that include the component	Correlations of indicators of the component with other wisdom measures
Desire for Understanding	Curious (Holliday & Chandler, 1986) Intellectual (Holliday & Chandler, 1986) Motivation to learn and grow throughout the life course (König & Glück, 2013) Passion for truth and knowledge (Yang, 2001) Search/desire to understand (Kałużna-Wielobób, 2014) Well-read (Holliday & Chandler, 1986)	***Berlin Wisdom Model*:** “like any expertise, the acquisition and refinement of wisdom involves an extended and intense process of learning, practice, as well as the motivation to strive toward excellence” (Baltes & Staudinger, 2000, p. 127) ***HERO(E) Model of Wisdom*:** “By seeking to understand and derive insight from both our mistakes and successes, we are better prepared to confront similar issues in the future. Wise persons achieve a more balanced perspective on difficult life matters, hone a set of relevant coping skills, and reinforce an evolving sense of self-efficacy in relation to landmark events.” (Webster, 2007, p. 168) ***MORE Life Experience Model*:** “Exploratory processing [as part of the reflectivity resource] is an analytical and interpretive way of reflecting about life events that emphasizes meaning-making (i.e., extracting lessons and insights), complexity, and growth from the past” (Glück et al., 2019, p. 362) ***Three-Dimensional Wisdom Model*:** [The affective/compassionate dimension describes a desire] “to comprehend the significance and deeper meaning of phenomena and events, particularly with regard to intrapersonal and interpersonal matters” (Ardelt, 2003, p. 278)	Broader personality-growth factor: BWP: *r* = .55** (Wink & Staudinger, 2016) BWP: *r* = .28** (Mickler & Staudinger, 2008) Cognitive dimension of the 3DWS: affective dimension: *r =* .30** (Ardelt, 2003) reflective dimensions *r =* .41** (Ardelt, 2003) Intellect/Seek: SWIS: *r* = .23** (Brienza et al., 2018) Life insight (as a value): BWP: *r* = .15*, controlling for 7 covariates (Kunzmann & Baltes, 2003) SAWS: *r* = .51** (Webster, 2010) Personal growth: ASTI: *r* = .22** (Glück et al., 2013) BWP: *r* = .29 ** (Staudinger et al., 1997) SAWS: *r* = .51** (Ardelt, 2011) SAWS: *r* = .28** (Glück et al., 2013) 3DWS: *r* = .52** (Ardelt, 2011) 3DWS: *r* = .41** (Glück et al., 2013) Psychological-mindedness: BWP: *r* = .28** (Staudinger et al., 1997) Search for meaning: SAWS: *r* = .16** (Webster et al., 2017)
Open-mindedness	Accepting manner toward others (Holliday & Chandler, 1986) Flexibility (König & Glück, 2013) Flexible (Holliday & Chandler, 1986) Open to learn from others (Sternberg, 1985) Open-minded (Holliday & Chandler, 1986) Openness (Kałużna-Wielobób, 2014) Openness (König & Glück, 2013) Respecting (Holliday & Chandler, 1986) Tolerant of others’ faults and shortcomings (Hu et al., 2018)	***HERO(E) Model of Wisdom*:** “an openness to alternate views, information, and potential solution strategies optimizes the wise person’s effort to surmount obstacles efficiently. Exploring possibilities, entertaining discordant opinions, and investigating novel approaches to ongoing conundrums builds a repertoire of skills from which the wise person can draw when confronting life’s challenges. Openness, as applied to wise persons, concerns not only an orientation to the press and strains of the external world, but also to the interior landscape of mental life.” (Webster, 2007, p. 166) ***MORE Life Experience Model*:** “Wise individuals are interested in viewing situations from multiple perspectives [. . .]. They are non-judgmental, accept goals and values that differ from their own, and enjoy learning from others. They seek out new experiences and adapt well to the changes life inevitably brings.” (Glück et al., 2019, p. 350).	Big Five openness: ASTI: *r* = .20** (Levenson et al., 2005) ASTI: *r* = .44** (Glück et al., 2013) BWP: *r* = .20** (Glück & Baltes, 2006) BWP: *r* = .37** (Glück et al., 2013) BWP: *r* = .31** (Pasupathi & Staudinger, 2001) BWP: *r* = .42** (Staudinger et al., 1997) BWP: *r* = .25** (Staudinger et al., 1998) BWP: *r* = .20** (Glück & Baltes, 2006) BWP: *r* = .47** (adolescents): *r* = .23** (adults) (Staudinger & Pasupathi, 2003) BrWP: *r* = .27** (Mickler & Staudinger, 2008) SAWS: *r* = .41** (Glück et al., 2013) SAWS: *r* = .46** (Webster et al., 2014) SWIS: *r* = .19** (Brienza et al., 2018) 3DWS: *r* = .59** (Glück et al., 2013) Openness component of the MORE Life Experience Interview: correlations with the other components *r* = .18 to .64** (Glück et al., 2019) Self-emotions appraisal: SWIS: *r* = .10** (Brienza et al., 2018) 3DWS: *r* = .39** (Zacher et al., 2013)
Empathic Concern	Compassionate (Holliday & Chandler, 1986; Yang, 2001) Compassionate relationships (Montgomery et al., 2002) Empathetic (Clayton & Birren, 1980; Glück & Bluck, 2011; König & Glück, 2013; Holliday & Chandler, 1986) Sensitive (Holliday & Chandler, 1986) Sincere and warm-hearted (Yang, 2001) Sympathetic (Hershey & Farrell, 1997) Understanding (Clayton & Birren, 1980; König & Glück, 2013) Warm-hearted (König & Glück, 2013)	***MORE Life Experience Model*:** “We view empathy as an important precondition for the development of wisdom: those able to take others’ perspectives are more likely to develop a view of life that takes the needs of others and the common good into account” (Glück et al., 2019, p. 351) Emotional sensitivity “refers to an individual’s interest in and ability to identify the emotions experienced and expressed by him- or herself and others” (Kunzmann & Glück, 2019, p. 590) ***Three-Dimensional Wisdom Model*:** [The affective/compassionate dimension describes] “the presence of positive emotions and behavior toward other beings, such as feelings and acts of sympathy and compassion, and the absence of indifferent or negative emotions and behavior toward others” (Ardelt, 2003, pp. 278–279).	Empathy component of the MORE Life Experience Interview: *r* = .37** to .59** with the other components (Glück et al., 2019) IRI Empathic Concern: ASTI: *r* = .28** (Glück et al., 2013) BWP: *r* = -.01 (Glück et al., 2013) SAWS: *r* = .39** (Glück et al., 2013) 3DWS: *r* = .26** (Glück et al., 2013) Others’ emotions appraisal: SWIS: *r* = .21** (Brienza et al., 2018) 3DWS: *r* = .37** (Zacher et al., 2013)
Common-Good orientation	Benevolent (Yang, 2001) Care for others (Kałużna-Wielobób, 2014) Charitable (Hershey & Farrell, 1997) Concern for others (Sternberg, 1985) Ethical (Hershey & Farrell, 1997; König & Glück, 2013) Fair (Hershey & Farrell, 1997; Holliday & Chandler, 1986; Sternberg, 1985) Good-hearted (Yang, 2001) Kind (Holliday & Chandler, 1986) Love for humanity (Glück & Bluck, 2011) Love for others (Hu et al., 2018) Moral principles (Montgomery et al., 2002) Moral sensitiveness König & Glück, 2013 Orientation toward goodness (Glück & Bluck, 2011) Unselfish (Holliday & Chandler, 1986)	***Balance Theory of Wisdom*:** “wisdom is defined as the application of tacit knowledge as mediated by values toward the achievement of a common good” (Sternberg, 1998, p. 347) ***Berlin Wisdom Model*:** “Wisdom, of course, is not meant to imply full-blown relativity of values and value-related priorities. On the contrary, it includes an explicit concern with the topic of virtue and the common good.” (Baltes & Staudinger, 2000, p. 126) ***Common Wisdom Model*:** “By moral grounding we mean a set of inter-related aspirational goals: balance of self- and other-oriented interests, pursuit of truth (vs. dishonesty), and orientation toward shared humanity.” (Grossmann, Weststrate, Ardelt, et al., 2020, p. 133) ***Self-Transcendence Model*:** Self-transcendent individuals are “able to dissolve rigid boundaries between themselves and others, truly care about others, and feel that they are part of a greater whole” (Koller et al., 2017, p. 4)	Benevolence: ASTI: *r* = .22**(Glück et al., 2020) 3DWS: *r* = .26** (Glück et al., 2020) Communal relationship orientation: SWIS: *r* = .24** (Brienza et al., 2018) Ecological protection value: BWP: *r* = .13* (controlling for 7 covariates) (Kunzmann & Baltes, 2003) SAWS: *r* = .39** (Webster, 2010) Generativity: BWP: *r* = .34** (latent variables) (Wink & Staudinger, 2016) SAWS: *r* = .44** (Webster, 2003) SAWS: *r* = .45** (Webster, 2007) Societal engagement value: BWP: *r* = .14* (controlling for 7 covariates) (Kunzmann & Baltes, 2003) SAWS: *r* = .26** (Webster, 2010) Universalism: ASTI: *r* = .26**(Glück et al., 2020) 3DWS: *r* = .24**(Glück et al., 2020) Well-being of friends value: BWP *r* =.17* (controlling for 7 covariates) (Kunzmann & Baltes, 2003) SAWS *r* =.34** (Webster, 2010)
Emotion regulation	Calm (Hershey & Farrell, 1997) Composure (Kałużna-Wielobób, 2014) Cool and calm (Yang, 2001) Does not anger easily (Brezina, 2010) Emotional control (Chen et al., 2014) Emotions under control (Brezina, 2010) Even-tempered (Holliday & Chandler, 1986) Gentle (Clayton & Birren, 1980) Holliday & Chandler, 1986 Humor (König & Glück, 2013) Inner peace (König & Glück, 2013) Inner stability (König & Glück, 2013) Patience (Kałużna-Wielobób, 2014) Patience (Holliday & Chandler, 1986; König & Glück, 2013) Peaceful (Brezina, 2010; Clayton & Birren, 1980; Hershey & Farrell, 1997) Poised (Holliday & Chandler, 1986) Quiet (Hershey & Farrell, 1997; Holliday & Chandler, 1986) Regulation of self-emotion (Hu et al., 2018) Relaxed (Holliday & Chandler, 1986) Remains calm under pressures (Brezina, 2010) Socially unobtrusive (Holliday & Chandler, 1986)	***Bremen Wisdom Model*:** “The second basic criterion is that a self-wise person knows heuristics of growth and self-regulation (e.g., how to express and regulate emotions or how to develop and maintain deep social relations). Humor is an example of an important heuristic that helps one to cope with various difficult and challenging situations and to learn from them at the same time.” (Mickler & Staudinger, 2008, p. 788) ***HERO(E) Model of Wisdom*:** “the emotional dimension of wisdom involves an exquisite sensitivity to the gross distinctions, subtle nuances, and complex blends of the full range of human affect. Recognizing, embracing, and employing emotions in a constructive and creative way are a benchmark of wisdom. Such appropriate use of emotions concerns the entire panoply of affective valence, spanning the spectrum from rage, grief, and frustration to happiness, joy, and ecstasy.” (Webster, 2007, p. 166) ***MORE Life Experience Model*:** “Wise individuals are attentive to their emotions, tolerant of ambivalent feelings, and able to manage emotion as fits the situation . . . As their aim is to understand life more fully, wise individuals neither suppress negative feelings nor dwell on them extensively . . .” (Glück et al., 2019, p. 351)	Emotional competence/self: 3DWS: *r* = .63** (Glück et al., 2013) ASTI: *r* = .50** (Glück et al., 2013) BWP: *r* = .28** (Glück et al., 2013) SAWS: *r* = .32** (Glück et al., 2013) Emotional competence/others: 3DWS: *r* = .48** (Glück et al., 2013) ASTI: *r* = .47** (Glück et al., 2013) BWP: *r* = .27** (Glück et al., 2013) SAWS: *r* = .45** (Glück et al., 2013) Emotion(al) regulation: SWIS: *r* = .12** (Brienza et al., 2018) 3DWS: *r* = .35** (Ardelt, 2011) 3DWS: *r* = .40** (Zacher et al., 2013) Emotion Regulation component of the MORE Life Experience Interview: *r* = .41** to .59** with the other components (Glück et al., 2019) Emotion regulation/Reappraisal: SWIS *r* = .23** (Brienza et al., 2018)
Life Knowledge	Answers to fundamental questions of life (Hu et al., 2018) Broad spectrum of positive and negative experiences (Glück & Bluck, 2011) Experienced (Clayton & Birren, 1980; Holliday & Chandler, 1986; Sternberg, 1985) Knowledge and insight (Chen et al., 2014) Knowledge and life experience (Glück & Bluck, 2011) Knowledge of people/human nature (Kałużna-Wielobób, 2014) Knowledge of the world (Kałużna-Wielobób, 2014) Knowledgeable (Clayton & Birren, 1980; König & Glück, 2013) Learns from experience (Holliday & Chandler, 1986; Sternberg, 1985; Yang, 2001) Life experience (Kałużna-Wielobób, 2014; König & Glück, 2013) Life wisdom (Kałużna-Wielobób, 2014) Understands life (Holliday & Chandler, 1986)	***Balance Theory of Wisdom*:** “In the balance theory, wisdom is viewed as the use of TK [tacit knowledge] as mediated by positive ethical values toward the common goal of attaining a common good via a balance among multiple [interests and responses to environmental context].” (Sternberg, 2019, p. 165) ***Berlin Wisdom Model*:** “The factual knowledge part concerns knowledge about such topics as human nature, life-long development, variations in developmental processes and outcomes, interpersonal relations, social norms, critical events in life and their possible constellations, as well as knowledge about the coordination of the well-being of oneself and that of others. Procedural knowledge about the fundamental pragmatics of life involves strategies and heuristics for dealing with the meaning and conduct of life—for example, heuristics for giving advice and for the structuring and weighing of life goals, ways to handle life conflicts and life decisions, and knowledge about alternative back-up strategies if development were not to proceed as expected.” Baltes & Staudinger, 2000, p. 125 ***HERO(E) Model of Wisdom*:** “consequential events of both a positive and negative nature profoundly shape and enrich psychological growth and development. Nevertheless, it is those problematic and disturbing episodes in our lives which seem to capture our attention and engage people with their demand for meaning-making for longer and more intense periods of reflection” (Webster, 2007, pp. 167–168) ***Three-Dimensional Wisdom Model*:** “Intellectual or theoretical knowledge is knowledge that is understood only at the intellectual level, whereas wisdom is understood at the experiential level. It is only when an individual realizes (i.e., experiences) the truth of this preserved knowledge that the knowledge is re-transformed into wisdom and makes the person wise(r). If the truth is only understood intellectually, it remains intellectual (theoretical) knowledge and does not lead to a personality transformation of the individual” (Ardelt, 2004, p. 260)	Factual Knowledge component of the BWP, correlations with the other BWP criteria: *r* = .70** to .75** (Baltes et al., 1995) *r* = .48**to .62** (Mickler & Staudinger, 2008) *r* = .38** to .68** (Glück et al., 2019) Procedural Knowledge component of the BWP, correlations with the other BWP criteria: *r* = .55** to .80** (Baltes et al., 1995) *r* = .35** to .50** (Mickler & Staudinger, 2008) *r* = .48** to .73** (Glück et al., 2019) Number of life events reflected upon predicts increases in wisdom-related performance after a wisdom instruction, β = .19** (Glück & Baltes, 2006)
Self-knowledge	Knowing oneself (Hu et al., 2018) Knows self best (Sternberg, 1985) Self-awareness (Kałużna-Wielobób, 2014) Understands self (Holliday & Chandler, 1986)	***Bremen Wisdom Model*:** “The first basic criterion is rich self-knowledge, that is, deep insight into oneself and one’s own life. A self-wise person is aware of his or her own competencies and weaknesses, emotions, and goals and has developed a sense of meaning in life.” (Mickler & Staudinger, 2008, p. 788) ***Self-Transcendence Model*:** “Self-knowledge is the awareness of the sources of one’s sense of self. The sense of self arises in the context of roles, achievements, relationships, and beliefs. It is also a sense of enduring duality that we conceptualize as self and other.” (Levenson et al., 2005, p. 128)	Ego development: BrWP: *r* = .26** (Mickler & Staudinger, 2008) Ego integrity: SAWS: *r* = .23** (Webster, 2003) Informational identity style: ASTI: *r* = .39** (Beaumont, 2009) Self-concept maturity: BWP: *r* = .19* (Mickler & Staudinger, 2008) BrWP: *r* = .28** (Mickler & Staudinger, 2008) Self-Knowledge component of the BrWP: *r* = .31** to .57** with the other BrWP criteria (Mickler & Staudinger, 2008)
Awareness and Acceptance of Uncertainty and Uncontrollability	Acts within own limitations (Sternberg, 1985) Modest/humble (Holliday & Chandler, 1986) Cautious and modest (König & Glück, 2013) Humility (Kałużna-Wielobób, 2014) Humble (Yang, 2001) Offers alternative solutions to problems (Brezina, 2010)	***Berlin Wisdom Model*:** “. . ., the recognition of and management of uncertainty, is based on the ideas . . . that (a) the validity of human information processing itself is essentially limited (constrained), (b) individuals have access only to select parts of reality, and (c) the future cannot be fully known in advance. Wisdom-related knowledge and judgment are expected to offer ways and means to deal with such uncertainty about human insight and the conditions of the world, both individually and collectively.” (Baltes & Staudinger, 2000, p. 126) ***Bremen Wisdom Model*:** “ . . ., tolerance of ambiguity, involves the ability to recognize and manage the uncertainties in one’s own life and one’s own development. It means being aware of and able to deal with the fact that the present and the future are full of uncontrollable and unpredictable events, such as accidents and illnesses, and also that one’s past is never fully known.” (Mickler & Staudinger, 2008, p. 788) ***MORE Life Experience Model*:** “Wise individuals, however, are more realistically aware of the uncertainty and unpredictability of life . . . while also feeling that, having learned from experience, they will somehow be able to master whatever happens. Thus, mastery is a dialectical concept that combines full awareness of life’s uncontrollability and unpredictability with trust in one’s own ability to cope.” (Glück et al., 2019, p. 350) ***Three-Dimensional Wisdom Model*:** “[The cognitive dimension] includes knowledge of the positive and negative aspects of human nature, of the inherent limits of knowledge, and of life’s unpredictability and uncertainties.” (Ardelt, 2003, p. 278) ***Wise Reasoning Model*:** “epistemic humility (e.g., unbiased/accurate thinking, seeing through illusions, understanding one’s limitations)” (Grossmann, Weststrate, Ardelt, et al., 2020, p. 133) [Wisdom criteria:] “intellectual humility/recognition of limits of one’s own knowledge” . . .; “recognition of uncertainty and change” (Grossmann, 2017, p. 237)	Factor loadings on a common-factor model with three other wisdom components across several samples (Brienza et al., 2018): SWIS Intellectual humility: .74 to .85 SWIS Awareness of likelihood of change/ multiple outcomes: .72 to .84 Limits of knowledge component of the WRP: *r* = .03 to .43** with the other WRP components: (Grossmann et al., 2013) SWIS Intellectual humility: SAWS: *r* = .35** (Brienza et al., 2018) 3DWS: *r* = .15** (Brienza et al., 2018) ASTI: *r* = .03 (Brienza et al., 2018) SWIS Awareness of likelihood of change/ multiple outcomes: SAWS: *r* = .30** (Brienza et al., 2018) 3DWS: *r* = .14* (Brienza et al., 2018) ASTI: *r* = .17** (Brienza et al., 2018) Tolerance of Ambiguity Component of the BrWP: *r* = .38** to .54** with the other BrWP criteria (Mickler & Staudinger, 2008) Uncertainty component of the BWP, correlations with the other BWP criteria: *r* = .55** to .83** (Baltes et al., 1995) *r* = .40** to .60** (Mickler & Staudinger, 2008) *r* = .38** to .54** (Glück et al., 2019)
Awareness and Acceptance of Divergent Perspectives	Sees things within larger context (Holliday & Chandler, 1986) Sees and considers all points of view (Holliday & Chandler, 1986) Listens to all sides of an issue (Sternberg, 1985) Listens to all sides before deciding (Brezina, 2010) Tolerance (Kałużna-Wielobób, 2014; König & Glück, 2013) Acceptance of others’ perspectives and values (Glück & Bluck, 2011) Tolerant of others’ faults and shortcomings (Hu et al., 2018)	***Balance Theory of Wisdom*:** “Wisdom involves practical intelligence used in particular to achieve a balance of interpersonal, intrapersonal, and extrapersonal interests.” (Sternberg, 2019, p. 166) ***Berlin Wisdom Model*:** “The first metacriterion, lifespan contextualism, is meant to identify knowledge that considers the many themes and contexts of life (e.g., education, family, work, friends, leisure, the public good of society, etc.), their interrelations and cultural variations, and in addition, incorporates a lifetime temporal perspective (i.e., past, present, and future). Another feature of lifespan contextualism is the historical and social location of individual lifespan development as well as the idiographic or nonnormative events that operate in human development . . . The second wisdom-specific metacriterion, relativism of values and life priorities, deals with the acknowledgment of and tolerance for value differences and the relativity of the values held by individuals and society.” (Baltes & Staudinger, 2000, pp. 125–126) ***Bremen Wisdom Model*:** “[I]nterrelating the self, is characterized by an awareness of the contextual embeddedness of one’s behavior, feelings, or both. The contextualization can be threefold: age related, history related, and idiosyncratic. Interrelating the self also implies awareness of one’s biographical (diachronic) embeddedness, one’s dependency on others, and the interrelatedness of different self-domains. The second metacriterion is self-relativism. People high in self-relativism critically appraise their own behavior without losing a basic level of self esteem. They are able to tolerate others’ values as long as the balance between their own good and that of others is kept.” (Mickler & Staudinger, 2008, p. 788) ***Three-Dimensional Wisdom Model*:** “A deeper understanding of life is only possible if one can perceive reality as it is without any major distortions. To do this, one needs to engage in reflective thinking by looking at phenomena and events from many different perspectives to develop self-awareness and self-insight.” (Ardelt, 2003, p. 278) ***Wise Reasoning Model*:** “context-adaptability (e.g., practical or pragmatic reasoning, optimization of behavior to achieve certain outcomes), perspectivism (e.g., consideration of diverse perspectives, foresight and long-term thinking), dialectical and reflective thinking (e.g., balancing and integration of viewpoints, entertaining opposites),” (Grossman, Weststrate, Ardelt, et al., 2020, p. 133) [Wisdom criteria:] “Recognition of others’ perspectives/broader contexts than the issue at hand” . . .; “Integration of different opinions/preference for compromise” (Grossmann, 2017, p. 237)	Factor loadings on a common-factor model with three other wisdom components across several samples: SWIS Others’ perspectives: .75** to .83** (Brienza et al., 2018) SWIS Outsider’s vantage point: .47** to .73** (Brienza et al., 2018) Interrelating the Self component of the BrWP, correlations with the other BrWP criteria: *r* = .34**-.57** (Mickler & Staudinger, 2008) Lifespan Contextualism component of the BWP, correlations with the other BWP criteria: *r* = .58** to .80** (Baltes et al., 1995) *r* = .31** to .48** (Mickler & Staudinger, 2008) *r* = .47** to .74** (Glück et al., 2019) Perspective component of the WRP: *r* = 17**-.35** with the other WRP criteria: (Grossmann et al., 2013) Perspective-taking: SWIS: *r* =.48** (Brienza et al., 2018) Self-Relativism component of the BrWP, correlations with the other BrWP criteria: *r* = .31**-.57** (Mickler & Staudinger, 2008) SWIS Others’ perspectives: SAWS: *r* = .26** (Brienza et al., 2018) 3DWS: *r* = .17** (Brienza et al., 2018) ASTI: *r* = .22** (Brienza et al., 2018) SWIS Outsider’s vantage point: SAWS: *r* = .28** (Brienza et al., 2018) 3DWS: *r* = .01 (Brienza et al., 2018) ASTI: *r* = .13* (Brienza et al., 2018) Value Relativism component of the BWP, correlations with the other BWP criteria: *r* = .68** to .83** (Baltes et al., 1995) *r* = .31** to .62** (Mickler & Staudinger, 2008) *r* = .23* to .59** (Glück et al., 2019)
Self-Reflection	Ability to admit mistakes (Kałużna-Wielobób, 2014) Able to learn from one’s own mistakes (König & Glück, 2013) Being critical (König & Glück, 2013) Does not speak without considering words first (Brezina, 2010) Introspective (Clayton & Birren, 1980) Learning from mistakes (Kałużna-Wielobób, 2014) Learns from mistakes (Sternberg, 1985) Philosophical (Holliday & Chandler, 1986) Reflective (Hershey & Farrell, 1997) Reflective (Holliday & Chandler, 1986) Reflective attitude (Hu et al., 2018) Reflectiveness (Chen et al., 2014) Reflectiveness (König & Glück, 2013) Self-reflection and self-criticism (Glück & Bluck, 2011) Thinks before acting or speaking (Sternberg, 1985) Thoughtful/thinks a great deal (Holliday & Chandler, 1986) Unafraid to admit a mistake (Sternberg, 1985)	***Common Wisdom Model*:** “dialectical and reflective thinking (e.g., balancing and integration of viewpoints, entertaining opposites)” (Grossmann, Weststrate, Ardelt, et al., 2020, p. 133) ***HERO(E) Model of Wisdom*:** “By seeking to understand and derive insight from both our mistakes and successes, we are better prepared to confront similar issues in the future. Wise persons achieve a more balanced perspective on difficult life matters, hone a set of relevant coping skills, and reinforce an evolving sense of self-efficacy in relation to landmark events.” (Webster, 2007, p. 168) ***MORE Life Experience Model*:** “We define reflectivity as a person’s motivation to think about complex issues in a complex way. Reflective people look back on life experiences and think deeply about them. They are willing to question their own past and current views and behavior, as their goal is to develop a deeper understanding and not to reassure their own views.” (Glück et al., 2019, p. 351) ***Three-Dimensional Wisdom Model*:** “. . . reflective thinking by looking at phenomena and events from many different perspectives to develop self-awareness and self-insight. This practice will gradually reduce one’s self-centeredness, subjectivity, and projections, and increase one’s insight into the true nature of things, including the motivations of one’s own and other people’s behavior.” (Ardelt, 2003, p. 278)	Attributive complexity: SWIS: *r* = .22** (Brienza et al., 2018) Exploratory (growth-oriented) processing: self-report wisdom composite: β = .43** (Weststrate & Glück, 2017a) performance wisdom composite: β = .31** (Weststrate & Glück, 2017a) SAWS: *r* = .22** (Webster et al., 2017) Judicial cognitive style: BWP: *r* = .19* (Pasupathi & Staudinger, 2001) BWP: *r* = .25** (Staudinger et al., 1997) Perspective-taking: SWIS: *r* = .48** (Brienza et al., 2018) Reflective awareness: ASTI/SAWS composite: *r* = .66** (Verhaeghen, 2020) Reflectivity component of the MORE Life Experience Interview, correlations with the other components: *r* = .22* to .63** (Glück et al., 2019) Ruminative reflection: SWIS: *r* = .26** (Brienza et al., 2018)

*Note*. BWP = Berlin Wisdom Paradigm (Baltes & Staudinger, 2000); 3DWS = Three-Dimensional Wisdom Scale; SWIS = Situated Wise Reasoning Scale (Brienza et al., 2018); SAWS = Self-Assessed Wisdom Scale (Webster, 2007); ASTI = Adult Self-Transcendence Inventory (Levenson et al., 2005), BrWP = Bremen Wisdom Paradigm (Mickler & Staudinger, 2008); IRI = Interpersonal Reactivity Index (Davis, 1983); WRP = Wise-reasoning Paradigm (Grossmann et al., 2010).

* *p* < .05. **p < .01.

#### Consistency with experts’ conceptions of wisdom

Second, a model of wisdom should obviously be consistent with experts’ conceptions of wisdom. As we explained earlier, we aimed to integrate different definitions of wisdom in our model, including all components that contribute to wise behavior in real life. For this purpose, we drew on the psychological models of wisdom reviewed earlier; the second column of Table 3 shows which wisdom models include each component. In addition, we drew on research studying wisdom researchers’ conceptions of wisdom. Jeste et al. (2010) conducted a study on experts’ beliefs about the importance of specific characteristics and experiences to wisdom. They asked experts to rate the importance of 53 items for wisdom on a scale from 1 to 9. We report the ratings for the items corresponding to each component of our model in the following.

#### Consistency with empirical evidence

Third, the integrative wisdom model obviously needs to be consistent with empirical evidence. For each component of wisdom in our model, the third column of Table 3 reports relationships of measures of the respective construct with wisdom measures that do not include it as a component, as well as correlations of each component with the other components of the wisdom models that include it.

#### Thought experiments

Fourth, we used a thought-experimental approach to decide which components to include. Would, for example, a person be considered as wise if they showed a considerable amount of life knowledge, but little concern for others? Vice versa, would a person be considered as wise if they showed a great deal of concern for others but little knowledge about life? In both cases, the answer would be no, so we considered both life knowledge and concern for others as components of wisdom. As another example, would a behavior be considered as wise if it occurred completely intuitively without any use of reasoning and reflection? In that case, our answer was no, so while intuition might be relevant for some wise behaviors, it does not constitute a necessary component of wisdom. As another thought experiment, we asked: If we could increase a person’s level of a given characteristic through an intervention, would this person also become wiser? For example, would an increase in life knowledge, empathy, or intuition make a person wiser? If so, the characteristic was a candidate for inclusion in the model.

In the following, we present the integrative model of wise behavior. We first describe the structure of the model and then discuss each component in detail.

## The Integrative Wisdom Model

**Figure 1.**
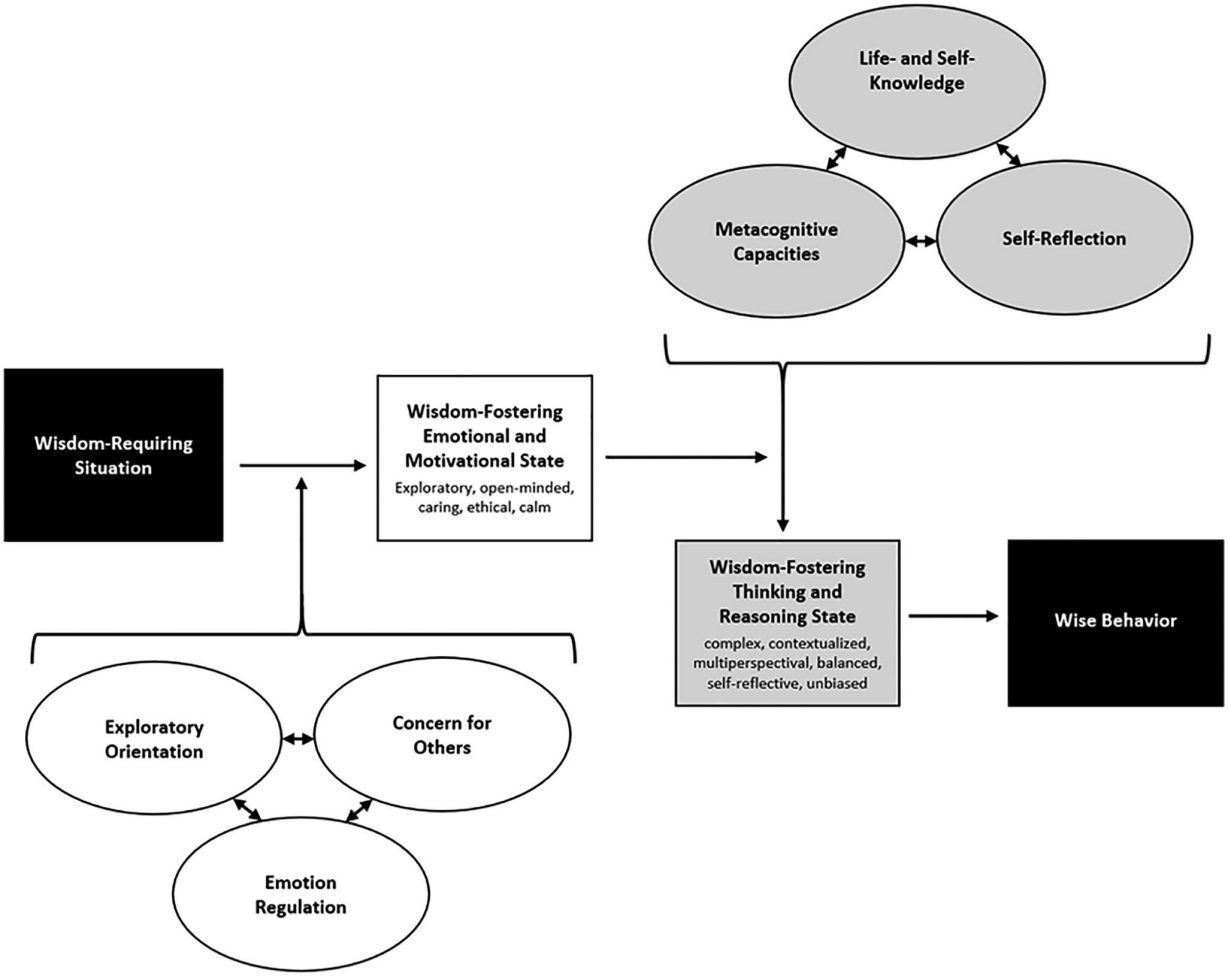
The structure of the integrative wisdom model.

**Figure 2.**
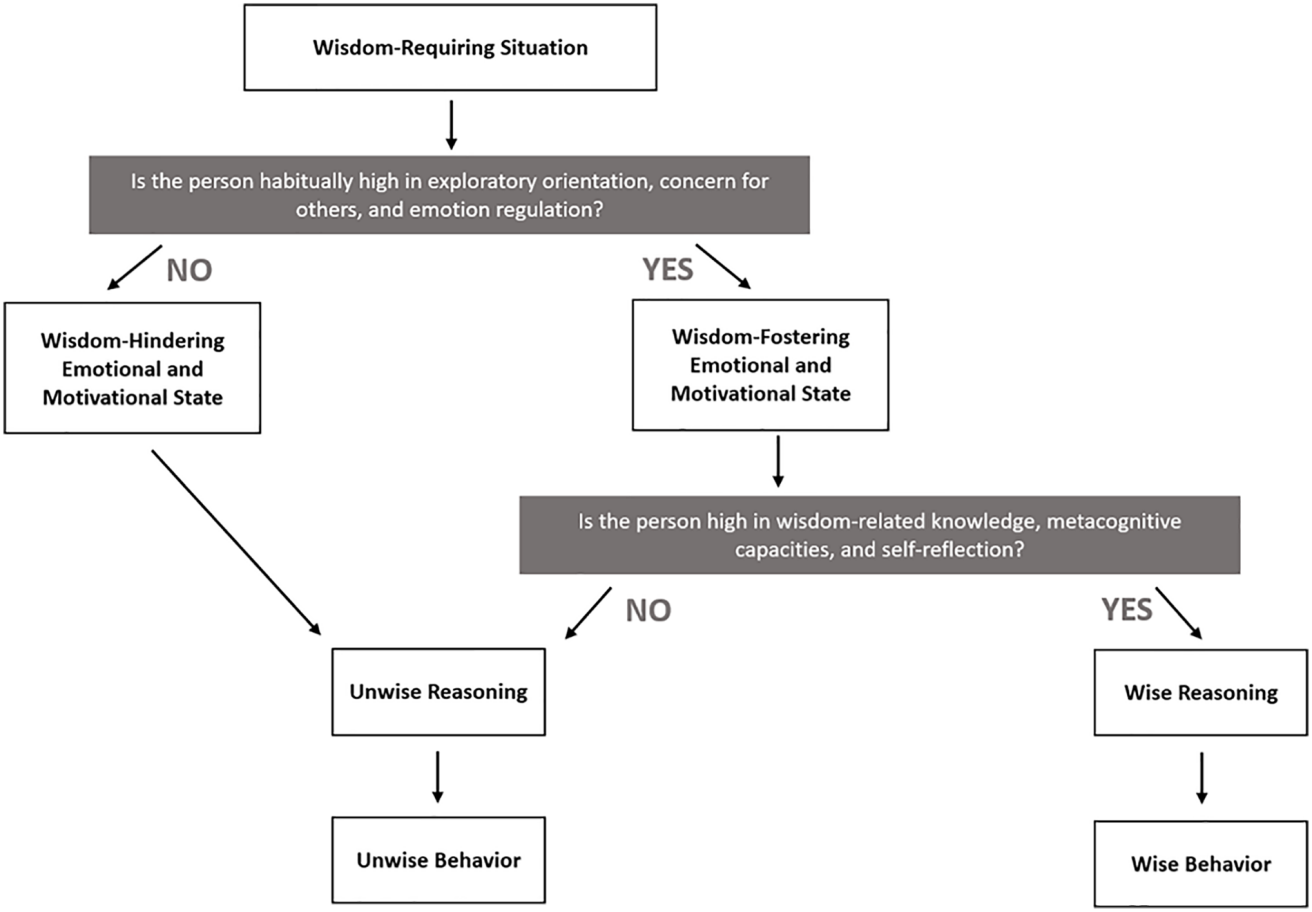
A tree-diagram depiction of the integrative wisdom model.

Figure 1 displays the structure of the Integrative Wisdom Model. Figure 2 represents the model as a tree diagram of pathways toward wise and unwise behavior. Consistent with recent research (overviews in Grossmann, 2017; Grossmann, Kung, & Santos, 2019), the model includes both situation-specific state and general trait components. In Figure 1, state variables are displayed as rectangles and include the wisdom-requiring situation, a person’s emotional and motivational state in that situation, the quality of their problem-based reasoning, and the resulting wise or less wise behavior. Trait variables, displayed as ellipses, are overarching cognitive and noncognitive characteristics that influence a person’s reactions to the situation.

### Overview of the Model

As Figure 1 shows, the model describes wise behavior in a wisdom-requiring situation, that is, a complex, uncertain, emotionally challenging real-life problem as discussed earlier. The problem evokes an emotional and motivational state that is influenced by trait characteristics of the individual—a person who is habitually exploration-oriented, concerned for others, and good at regulating their own and others’ emotions will be better able to remain open-minded, caring, and calm even in a challenging situation. For people low in any of these characteristics, the same situation may trigger high emotional arousal and/or a narrow focus on one’s own or one side’s interests rather than balancing all interests.

Perhaps the most important link in the model is that the person’s emotional and motivational state in the situation influences the extent to which they can draw on their wisdom-related knowledge, metacognitive capacities, and self-reflection. Wise individuals have ample knowledge about life and themselves, which is closely intertwined with their metacognitive capacities, enabling them to acknowledge different viewpoints, consider contextual and situational influences, and include uncertainty in their predictions (Baltes & Staudinger, 2000; Grossmann, 2017; Grossmann, Weststrate, Ardelt, et al., 2020). They are also able to reflect on their own thinking and make sure that their personal preferences and biases do not influence their judgment (Grossmann, Weststrate, Ardelt, et al., 2020; Weststrate & Glück, 2017a). They are able to do all this not just in a wisdom research lab but also in a real-life problem situation because their emotional and motivational state enables them to fully utilize their cognitive capacities. Therefore, they manage to act wisely and resolve the situation in a way that optimizes the common good and balances the needs of everyone involved (Sternberg, 2019).

In the following section, we discuss each component of the model, describing its relevance for wise behavior and the empirical evidence supporting its inclusion in the model. Table 3 displays the evidence for the inclusion of each component in detail, based on the inclusion criteria we discussed earlier: (a) consistency with non-experts’ conceptions of wisdom, (b) presence of the component in existing psychological wisdom models, and (c) empirical evidence for the relationship between the component and wisdom. Evidence from Jeste et al.’s (2010) expert survey will be reported in the text.

### Noncognitive Components: Exploratory Orientation, Concern for Others, and Emotion Regulation

As shown in Figure 1, how a person reacts to a wisdom-requiring problem—i.e., their emotional and motivational state—is influenced by three general non-cognitive traits: exploratory orientation, concern for others, and emotion regulation.

#### Exploratory orientation

Wise individuals have a basic motivation to understand life and to learn and grow from experiences. In difficult situations, they aim for a deep, complex, realistic, and illusion-free understanding of the problem. Therefore, they consider perspectives that differ from their own as interesting and informative, not as challenging or threatening. In the longer term, they grow in wisdom as they gain new insights from reflecting on experiences. Peterson and Seligman (2004) subsumed the character strengths of curiosity, open-mindedness, and love of learning under the virtue of “wisdom and knowledge.” The Integrative Wisdom Model distinguishes two related components that are particularly relevant for wise behavior in real life: a desire for understanding and open-mindedness.

##### Desire for understanding

Wise individuals show a deep curiosity about and fascination with the fundamental questions of the human existence (Ardelt, 2003). In concrete difficult situations, their curiosity motivates them to understand problems in depth, look at them from different perspectives, and consider contextual factors. As they reflect on their own and others’ experiences, they develop expertise on matters of human existence (Baltes & Staudinger, 2000; Glück, 2019a; Weststrate & Glück, 2017a).

While curiosity and a desire for understanding do not seem to be highly typical features of folk conceptions of wisdom, they were mentioned in several studies (see Table 3). As Table 1 shows, however, aiming for an in-depth understanding of a difficult situation was mentioned as a characteristic of wise *behavior* far more frequently than as a characteristic of wisdom as a trait. A desire for a deeper understanding of life is a component of the Three-Dimensional Wisdom Model (Ardelt, 2003); it is also implicated by the Berlin Wisdom Model’s definition of wisdom as expertise (Baltes & Staudinger, 2000) and the reflectivity components of the developmental wisdom models (Glück et al., 2019; Webster, 2007). In Jeste et al.’s (2010) Delphi study, wisdom experts rated “desire for learning/knowledge” at a mean of 8.0 on the 9-point scale of importance to wisdom. Empirically, several studies have found correlations of wisdom measures with indicators of a desire for understanding, such as valuing life insight, psychological mindedness, or a personal-growth orientation (see Table 3).

##### Open-mindedness

Wise individuals are open to beliefs and values that differ from their own, experiences that broaden their worldviews, and exploring inner experiences even if they are complex, ambivalent, or undesirable. In concrete difficult situations, their openness enables them to acknowledge, tolerate, and consider the divergent perspectives involved.

As Table 3 shows, openness, flexibility, and acceptance of others are part of folk conceptions of wisdom according to several studies. Openness is a component in both developmental models of wisdom, the HERO(E) model of wisdom (Webster, 2003, 2007), and the MORE Life Experience Model (Glück & Bluck, 2013). In the Delphi study of wisdom experts (Jeste et al., 2010), “openness to new experience” had an average rating of 8.2 of 9 for its importance to wisdom. Empirically, the Big Five factor of openness to experience is among the strongest and most consistent noncognitive predictors of various wisdom measures; correlations have also been found between wisdom and appraisal of one’s own emotions.

In sum, wise individuals are curious about life and oriented toward learning and growth, and they are open to new ideas, perspectives, and inner and outer experiences. These general traits enable them to react to difficult situations in an understanding-oriented and open-minded way.

#### Concern for others

The second noncognitive domain that we consider necessary for wisdom is a concern for others. Wise individuals are able to understand how others feel, and they care about the well-being of others. They aim to resolve difficult situations in ways that balance gains and losses for everyone involved, from the small scale of advice-giving to the large scale of social or political engagement.

##### Empathic concern

Empathic concern, the willingness and ability to accurately identify the emotions of others and experience sympathy with them, is a core component of wisdom. The ability component of empathy—being able to accurately infer someone else’s feelings—gives individuals access to relevant information about the situation and the people involved (Kunzmann & Glück, 2019). However, the affective component of empathy—sharing another person’s feelings—is not necessarily conducive to wisdom, as it may limit a person’s ability to see the broader picture and consider other perspectives (Bloom, 2016). Stange (2006) found that advice-givers in videos were judged as wisest if they displayed an intermediate amount of empathy—neither too little nor too much.

Compassion, empathy, and understanding are quite frequently part of folk conceptions of wisdom, as Table 3 shows. Empathic concern is a component of two wisdom models. The Three-Dimensional Wisdom Model (Ardelt, 2003) defines the affective dimension of wisdom as compassionate love for others. The MORE Life Experience model includes empathetic concern for others as a developmental resource for wisdom (Glück & Bluck, 2013; Glück et al., 2019). Empathy had a mean of 8.3 on the 9-point scale of importance for wisdom in Jeste et al.’s (2010) Delphi study. Relatively little research has looked at empirical relationships of empathy or compassion with measures of wisdom; so far, significant correlations have been reported with self-report measures but not performance measures of wisdom (see Table 3).

##### Common-good orientation

Wisdom entails a concern for “something larger” than one’s own benefit. Wise individuals are generally motivated to support others in need and to resolve difficult situations in ways that balance gains and losses for everyone involved. Their benevolence is not limited to those who are close to them; they care for humanity and the world as a whole.

Typical wisdom exemplars have often engaged themselves for others and effected major positive changes (examples include Mohandas Gandhi, Nelson Mandela, Martin Luther King, Mother Teresa, or Jesus Christ; see Paulhus et al., 2002; Weststrate et al., 2016). In addition, benevolence and concern for others, fairness and ethicality, and love for humanity are frequent components of folk conceptions of wisdom (see Table 3). Compared with the strong presence of a common-good orientation in folk conceptions, it seems to be somewhat underrepresented in experts’ conceptions of wisdom. In Jeste et al.’s (2010) Delphi study, “altruism,” “other-centeredness,” and “generativity” had means between 7 and 8 on the 9-point scale; only the broader terms “ethical conduct” and a “sense of justice and fairness” had means above 8. The Balance Theory of wisdom views aiming for a common good as the key characteristic that distinguishes wisdom from mere practical intelligence (Sternberg, 1998, 2019). Empirically, other-oriented value orientations and generativity are correlated with several measures of wisdom.

In sum, as Figure 1 shows, wise individuals are both willing and able to understand and consider the feelings of others in difficult situations. They accurately interpret and understand other people’s emotional states and engage themselves for problem solutions that benefit everyone involved, humanity at large, and the whole world.

#### Emotion regulation

Wise people are able to maintain their emotional balance even in highly challenging situations. In conceptualizing this component, we considered distinguishing between equanimity, as a habitually low emotional arousability, and emotion regulation, as the ability to manage emotions. However, habitual equanimity is not part of any wisdom model and the evidence of its relationship with wisdom (e.g., correlations between wisdom and neuroticism) is inconsistent. Therefore, we do not assume that wise individuals are habitually calm but that they are highly skilled at regulating emotions as situations require.

Wise individuals are experts in recognizing, understanding, and regulating emotions in themselves and others. Even in highly challenging situations, wise individuals manage not to be distracted by anger, fear, or worry. Humor may be an example of a wisdom-related emotion-regulatory capacity (Mickler & Staudinger, 2008; Webster, 2003, 2007).

As Table 3 shows, emotion regulation, equanimity, calmness, and peace of mind are quite typical components of folk conceptions of wisdom. Emotion regulation is a component of both developmental wisdom models, the HERO(E) model, and the MORE Life Experience Model. In the Delphi study, “emotional regulation” had a mean of 8.0 on the 9-point scale (Jeste et al., 2010). Empirically, correlations between emotion regulation and various measures of wisdom have been found quite consistently (see Table 3).

#### The “wisdom state of mind”: Open, caring, and calm

As explained earlier, the integrative wisdom model proposes that the three non-cognitive components of wisdom—an exploratory orientation, concern for others, and emotion regulation—are particularly important in those difficult, uncertain, and emotionally challenging situations where wisdom is most needed. In such situations, many people’s regulatory capacities are overwhelmed, but highly wise individuals are able to maintain an open-minded, caring, and calm state of mind even under very challenging circumstances.

As shown in Figure 1, a core feature of the model is the moderating effect of the non-cognitive components of wisdom on the relationship between the cognitive components and behavior. Wise individuals’ “wisdom state of mind” enables them to fully utilize their wisdom-related knowledge, metacognitive capacities, and self-reflection even in highly challenging situations. This assumption is supported by experimental research showing that taking a mental “step back” from one’s personal perspective increased wise reasoning (Grossmann & Kross, 2014; Kross & Grossmann, 2012; Staudinger & Baltes, 1996). Also, Grossmann et al. (2016) found correlations between state-level wise reasoning and lower emotional reactivity, greater emotional complexity, and more reappraisal in the respective situations. Glück et al. (2019) found that openness, empathy, and emotion regulation coded from narratives of difficult conflicts were predictive of BWP scores.

In sum, we argue that individuals who are able to maintain a state of emotional balance, open-mindedness, and concern for others even in highly challenging situations are able to fully utilize their wisdom-related cognitive capacities. These capacities are discussed next.

### Cognitive Components: Knowledge, Metacognition, and Self-Reflection

The integrative wisdom model distinguishes three closely related types of cognitive capacities: knowledge about life and oneself, metacognitive capacities, and self-reflection. One somewhat surprising feature of the cognitive components of wisdom was that while they are strongly emphasized in both folk conceptions and expert models of wisdom, there is little empirical evidence of their relevance for wisdom. While, for example, numerous studies have related openness to measures of wisdom (see Table 3), no study has ever tested the assumption that life knowledge is actually related to wisdom. The theoretical support for the cognitive components, however, is strong and consistent.

#### Life knowledge and self-knowledge

Wisdom involves broad and deep knowledge about life and oneself. Importantly, wisdom-related knowledge is not necessarily assumed to be conscious and verbalizable; conceptions of wisdom as expertise or practical intelligence (Baltes & Staudinger, 2000; Sternberg, 1998, 2019) suggest that much of the knowledge that wise individuals have may be tacit, implicit, or automatized.

##### Life knowledge

Broad and deep knowledge about life is widely considered an essential foundation of wisdom. Through reflecting on their own and others’ experience, wise individuals have acquired expertise about what Baltes and colleagues called the fundamental pragmatics of life (e.g., Baltes & Staudinger, 2000).

As Table 3 shows, life experience and life knowledge may be the most typical component of folk conceptions of wisdom. Life knowledge is also part of numerous wisdom models, but wisdom researchers diverge somewhat on the extent to which life knowledge needs to be based on personal experience. While the Berlin Wisdom Model does not make this claim (Baltes & Kunzmann, 2004), Ardelt (2004) argued that only personal experience can lead to personal insights and wisdom. Developmental wisdom models (Glück et al., 2019; Webster, 2007) seem to take the middle ground, assuming that experience-based learning is important for acquiring wisdom, but growth-oriented individuals are also able to gain wisdom-related insights from vicarious experiences (Glück et al., 2019). In Jeste et al.’s (2010) Delphi study, “learning from experience” and “rich knowledge of life” had ratings of 8.2 and 8.4, respectively, on the 9-point scale. As mentioned earlier, the idea that wisdom involves life knowledge seems to be so deeply ingrained in both non-experts’ and experts’ conceptions about wisdom that it has hardly been explicitly tested (see Table 3). Studies have found, however, that wiser individuals reflect more on life experiences and gain more insights from them (Glück & Baltes, 2006; Mickler & Staudinger, 2008; Weststrate, 2019; Weststrate & Glück, 2017a).

##### Self-knowledge

Wise people know a lot about life in general and about other people, but they also know a lot about themselves—their own personality, strengths, weaknesses, and needs. As part of their self-knowledge, they are more aware of their own biases and blind spots than other people, which helps them to gain a more objective, self-decentered perspective on problems.

As Table 3 shows, self-knowledge is not a particularly typical component of folk conceptions of wisdom. It is, however, an explicit part of Mickler and Staudinger’s (2008) model of personal wisdom and Levenson et al.’s (2005) conception of wisdom as self-transcendence (see also Yang, 2008). In addition, several authors have related wisdom to psychological maturity and high levels of ego development (Aldwin et al., 2019; Ardelt, 2019; Levenson et al., 2005; Mickler & Staudinger, 2008; Staudinger & Glück, 2011). In the Delphi study (Jeste et al., 2010), the item “self-insight” received a high expert rating of 8.6. As with life knowledge, relationships between self-knowledge and wisdom have not been studied directly, but Table 3 reports correlations between maturity and wisdom.

We consider life knowledge and self-knowledge as closely interrelated. People apply insights they gain about themselves to other people (sometimes leading to insights about differences between themselves and others), and they apply insights about life in general to themselves and their own life. As we discuss in the next section, wisdom-related knowledge is also translated into metacognitive capacities.

#### Metacognitive capacities

It is difficult to draw a clear distinction between the knowledge-related and metacognitive components of wisdom. The Berlin Wisdom Model, for example, describes awareness of the uncertainty, relativity, and contextuality of life experiences as components of wisdom-related knowledge, but it uses participants’ reasoning about concrete life problems to evaluate these components (Baltes & Staudinger, 2000). In fact, Grossmann and colleagues’ metacognitive criteria for wise *reasoning*, such as awareness of the limitations of one’s knowledge and consideration of broader contexts and others’ perspectives (e.g., Grossmann, 2017), derive quite directly from the criteria for wisdom-related *knowledge* in the Berlin model. In the following, we describe the two broad metacognitive capacities that are most essential to wise reasoning.

##### Awareness and consideration of uncertainty and uncontrollability

Wise individuals are keenly aware of the limitations of their own knowledge and power. They know that no one can predict the outcome of highly complex situations with absolute certainty, that unexpected things can happen to everyone at any time, and that much of what happens in our lives cannot be controlled.

Folk conceptions of this component center on the idea that wise individuals show humility (see Table 3). Awareness of uncertainty and unpredictability is a key component of models of wisdom-related knowledge and wise reasoning, and awareness of uncontrollability, that is, the limitations of one’s personal power to control or change the course of events, is part of Mickler and Staudinger’s Bremen model of personal wisdom a. In the Delphi study of experts’ conceptions of wisdom (Jeste et al., 2010), “recognizing the limits of one’s own knowledge” received a very high rating (8.8), while “humility” had a mean of 7.7. Concerning empirical evidence, Brienza et al. (2018) reported correlations of the SWIS components “intellectual humility” and “awareness of the likelihood of change” with other self-report measures of wisdom. Also, several studies showed that awareness of uncertainty loaded on one factor with other components of wisdom (see Table 3).

##### Awareness and consideration of divergent perspectives

The willingness and ability to consider and accept different perspectives, values, and goals is an important capacity of wise individuals. They are fully aware of how much life contexts and experiences shape people’s perspectives.

As Table 3 shows, folk conceptions of wisdom include wise individuals’ willingness to listen to every side of issues and to accept other perspectives. Awareness and acceptance of divergent perspectives are also components of several wisdom models. The Berlin Wisdom Model distinguishes value relativism, the acceptance of the multiplicity of values and priorities, from lifespan contextualism, the awareness and consideration of how life phases, contexts, and situations shape people’s interests and views. In Grossmann’s wise-reasoning research, wisdom criteria vary somewhat across studies, but consideration of different perspectives and contexts is always included (overview in Grossmann, 2017). According to Sternberg’s Balance Theory of Wisdom, a core characteristic of wise solutions to complex problems is that they balance divergent intra-, inter-, and extrapersonal interests (Sternberg, 1998). In the Delphi study of experts’ conceptions of wisdom (Jeste et al., 2010), “value relativism” had a mean of 8.2, and “tolerance of differences among others” had a mean of 8.5 on the 9-point scale. Similar to the uncertainty component, however, empirical evidence is mostly limited to relationships between components of wisdom models.

#### Self-reflection

Wise individuals are willing and able to reflect on their own feelings, thoughts, and behaviors, aiming to overcome blind spots and self-serving biases and to gain self-insight and self-knowledge. They acknowledge and reflect on their own emotions and intuitions so as to be guided but not controlled by them.

Being reflective and able to learn from mistakes are key components of folk conceptions of wisdom, as Table 3 shows. Aspects of reflectivity are also part of several wisdom models. The reflectivity components of the Three-Dimensional Wisdom Model (Ardelt, 2003) and the Common Wisdom Model (Grossmann, Weststrate, Ardelt, et al., 2020) focus on a willingness to self-decenter and consider different perspectives, whereas the developmental models emphasize self-insight from reflecting on experiences (Glück & Bluck, 2013; Glück et al., 2019; Webster, 2003, 2007). In the Delphi study, “self-reflection” was among the characteristics with the highest-rated importance to wisdom (8.6 out of 9; Jeste et al., 2010). Empirically, as Table 3 shows, various indicators of reflectivity, ranging from attributive complexity and a judicial cognitive style to exploratory reflection in autobiographical narratives, have been found to correlate with wisdom.

#### Wise reasoning and wise behavior

To summarize, their wisdom-fostering emotional and motivational state of mind enables wise individuals to utilize their life and self-knowledge in reasoning about life problems, to consider different perspectives and contextual factors and to not overestimate their knowledge or control over what happens, and to reflect on their own thinking and behavior without self-serving biases and blind spots. Therefore, they are able to think and act wisely: to gain comprehensive in-depth knowledge about the problem, to consider different pathways toward possible solutions, and to implement those pathways in close interaction with everyone involved. In addition to enabling wise reasoning, the noncognitive components of wisdom also contribute directly to wise behavior, which involves remaining open to everyone’s perspective, working toward a common good, and recognizing and regulating emotions of oneself and others.

To summarize, the Integrative Wisdom Model proposes that in highly challenging life situations, the noncognitive trait components of wisdom (exploratory orientation, concern for others, and emotion regulation) enable individuals to remain in an open-minded, caring, and calm mindset. Therefore, they are fully able to access and utilize their cognitive wisdom resources—broad and deep knowledge about life and themselves, metacognitive awareness of the limitations of knowledge and the relativity of perspectives, and self-reflection to reason and behave wisely in challenging situations. We next discuss the implications of the model.

## Implications of the Integrative Wisdom Model

As mentioned at the beginning of this article, the integrative wisdom model offers new perspectives on some current issues in wisdom research. We believe that the integrative wisdom model can explain the situation-specificity of wisdom, the relationship between personal and general wisdom, and the inconsistent relationships among measures of wisdom and between wisdom and other variables. The integrative model also has implications for the design of wisdom-fostering interventions and new measures of wisdom.

### Implications for Understanding the Situational Variability of Wisdom

As discussed earlier, wisdom varies considerably among individuals across situations (overview in Grossmann, 2017; Grossmann, Kung, & Santos, 2019)—most of us have had our wise moments as well as our very unwise ones. Four lines of evidence demonstrate the intraindividual variability of wisdom. First, experimental manipulations that lead individuals to de-center their perspective, such as having an imaginary conversation with someone about a problem before responding to it (Staudinger & Baltes, 1996), thinking about an issue from a geographically distant perspective (Kross & Grossmann, 2012), or imagining that a problem concerns someone else instead of oneself (Grossmann & Kross, 2014) can increase wise reasoning significantly. Second, even people who score low in measures of wisdom can recall situations in which they did something wise (Bluck & Glück, 2004; Glück et al., 2005). Third, wise reasoning varies from day to day (Grossmann et al., 2016). Fourth, wisdom varies even between narratives about autobiographical life challenges collected from the same individuals (Glück et al., 2019).

In other words, wisdom varies intraindividually across situations and even across memories. This variation cannot be due to situational variation in the cognitive components of wisdom: the wisdom-related knowledge we have accumulated in our life course and our metacognitive and self-reflective capacities should not vary on a short-term scale; we do not gain or lose them from one situation to the next. However, stored knowledge is not always equally accessible. The integrative wisdom model implies that the situational variability of wisdom is due to variability in the noncognitive components of wisdom.

Emotional and motivational states influence whether we can and want to access our wisdom-related knowledge. In relatively easy, unimportant, and emotionally unchallenging situations, many people are calm, friendly, and attentive to others’ perspectives. In more challenging situations, differences between people become more pronounced. Sometimes, people are not interested in understanding the depths of a complex situation; they prefer a simple and biased view. A focus on their own or their group’s benefit may lead people to ignore the needs of other people or groups. High emotional arousal can render people unwilling and unable to consider different perspectives. Thus, the extent to which people utilize their wisdom-related thinking capacities in a given situation depends on the extent to which they are in the wisdom-fostering mindset, which, according to the Integrative Wisdom Model, depends on their levels of the non-cognitive components of wisdom. Models that only look at wise reasoning cannot really explain why people sometimes seem to lose their wisdom-related cognitive capacities.

### Implications for Understanding the Difference Between Personal and General Wisdom

We believe that the integrative wisdom model can also account for the conceptual and empirical differences between what Staudinger and colleagues have called personal and general wisdom (Staudinger et al., 2005; see also Mickler & Staudinger, 2008; Staudinger, 2019; Staudinger & Glück, 2011). According to Staudinger (2019), the difference between general and personal wisdom is in how people think about others and about themselves. While we think about other people’s problems from a third-person perspective, our ability to look at ourselves “from the outside” is limited—when it comes to our own behavior, we all have considerable blind spots and biases. Therefore, according to Staudinger (2019), the two forms of wisdom do not necessarily coincide; a person can be high in general but low in personal wisdom. The classical example would be an excellent psychotherapist whose personal life is in disarray.

Based on the integrative wisdom model, we propose to view personal and general wisdom not as two qualitatively different forms of wisdom but as the poles of a continuum of personal involvement in a situation. With higher personal involvement in a problem, the importance of openness, concern for others, and emotion regulation for wise behavior increases. It is far easier to maintain one’s peace of mind and empathetic concern when one listens to a client’s story about her conflict with her partner than if one is in a conflict with one’s own partner, and it is even easier to talk about a fictitious person’s conflict in a psychological study. Thus, again, the noncognitive components of the integrative wisdom model can explain why people can be highly wise about a stranger’s problem and much less wise if the same problem occurs in their own life.

In addition, we consider it as unlikely that the two forms of wisdom are completely unrelated. First, as discussed earlier, self-knowledge and life knowledge are interrelated. Self-knowledge may help individuals deal with other people’s problems, and life knowledge may help people deal with their own problems. Second, we do not think that *any* life problems can be resolved without the use of self-knowledge, self-reflection, and self-regulation. When a friend asks a wise person for advice, for example, the wise person would be aware of their own possible biases about the problem and use self-reflection to ensure that they are fully able to see the friend’s perspective and needs. In other words, we propose that there is a continuum rather than a dichotomy between personal and general wisdom and that the importance of the non-cognitive and self-related components of wisdom vary in their relevance depending on a problem’s location on the continuum.

### Implications for Understanding the Relationships Between Different Wisdom Measures

The low correlations between different wisdom models were one starting point for our development of the Integrative Wisdom Model. As Table 3 shows, different wisdom models focus on different components of the integrative model. Some of the models have few components in common, others share more components. Therefore, one could predict that empirical correlations between wisdom measures should be higher when they have more components of the integrative model in common. Table 4 shows how the components of existing measures of wisdom fit into the integrative model.

**Table 4.** Mapping Psychological Definitions of Wisdom Onto the Components of the Integrative Wisdom Model.

Wisdom model	Emotion regulation	Exploratory orientation	Concern for others	Life & self- knowledge	Metacognitive capacities	Self-reflection
Berlin Wisdom Model				X	X	
Bremen Wisdom Model	X	X		X	X	X
Contextualized Wise Reasoning Model					X	X
H.E.R.O.(E). Model of Wisdom	X	X		X		X
MORE Life Experience Model	X	X	X		X	X
Self-Transcendence Model	X		X	X		X
Three-Dimensional Wisdom Model		X	X		X	X

*Note.* Berlin Wisdom Model (Baltes & Staudinger, 2000), Bremen Wisdom Model (Mickler & Staudinger, 2008), Contextualized Wise Reasoning Model (Grossmann, 2017), H.E.R.O.(E.) Model of Wisdom (Webster, 2007), MORE Life Experience Model (Glück et al., 2019), Self-Transcendence Model (Levenson et al., 2005), and Three-Dimensional Wisdom Model (Ardelt, 2003).

Few studies have used more than one measure of wisdom, but Table 5 shows the published correlations between measures and the number of components that the respective wisdom conceptions have in common. The size of the correlations corresponds quite well with the number of shared components; the Spearman correlation between the number of shared components and the z-transformed measure correlations was *r* = .78. Despite the fact that the correlations are also influenced by method variance, the number of components that two wisdom measures have in common in the integrative model accounts for a substantial part of the correlations between them. In other words, the integrative model can explain the low correlations between some measures of wisdom and the higher correlations between others.

**Table 5.** Numbers of Common Components, Empirical Correlations, and z-T﻿ransformed Correlations Between Measures of Wisdom.

Wisdom Measure	BrWP	3D-WS	ASTI	SAWS	SWIS
BWP No. of components shared Correlation	2 .48^1^ (*z* = .523)	1 .25^2^ (*z* = .255)	1 .30^2^ (*z* = .310)	1 .23^2^ (*z* = .234)	1 –
BrWP No. of components shared Correlation	-	3 –	3 –	4 –	2 –
3DWS No. of components shared Correlation		-	2 .58^2^ (*z* = .662)	2 .33^3^ *(z* = .343)	1 .21^4^ (*z* = .213)
ASTI No. of components shared Correlation			-	3 .50^2^ (*z* = .549)	1 .19^4^ (*z* = .192)
SAWS No. of components shared Correlation				-	1 .39^4^ (*z* = .412)

*Note.* BrWP = Bremen Wisdom Paradigm (Mickler & Staudinger, 2008); 3D-WS = Three-Dimensional Wisdom Scale; WP = Berlin Wisdom Paradigm (Baltes & Staudinger, 2000); ASTI = Adult Self-Transcendence Inventory (Levenson et al., 2005); SAWS = Self-Assessed Wisdom Scale (Webster, 2007); SWIS = Situated Wise Reasoning Scale (Brienza et al., 2018).

Superscript numbers refer to the publications from which the correlations were extracted:^1^ Mickler & Staudinger, 2008, ^2^ Glück et al., 2013, ^3^ Taylor et al., 2011, ^4^ Brienza et al., 2018.

### Implications for Understanding the Relationship Between Wisdom and Other Variables

The integrative wisdom model also has the potential to explain why different measures of wisdom sometimes have very different relationships with other variables (Glück, 2015). For example, measures that focus on noncognitive components of wisdom are more highly correlated with other noncognitive variables, such as value orientations (Glück et al., 2020; Kunzmann & Baltes, 2003; Webster, 2010) or well-being (overview in Ardelt, 2019), whereas measures that focus on cognitive components are more highly correlated with measures of intelligence and other cognitive capacities (Glück et al., 2013; Glück & Scherpf, in press; Grossmann et al., 2013; Staudinger et al., 1997).

As another example, the inconsistent relationships between wisdom and age are likely to be driven by the different wisdom measures’ different compositions of components. For example, studies have found a negative (Ardelt, 2003; Glück et al., 2013) or, more recently, an inverse U-shaped relationship (Ardelt et al., 2018) of the Three-Dimensional Wisdom Scale with age, suggesting that wisdom is highest in middle-aged participants and somewhat lower in older participants. This relationship is largely driven by the cognitive dimension of the scale, which assesses the “desire for understanding” component of the integrative wisdom model. Curiosity about life may decline with age in the general population, although highly wise individuals maintain high levels of it into old age (Ardelt et al., 2018; Glück, 2019a; Glück et al., 2013). Other components of wisdom, such as concern for others, may be positively related to age in the general population, and a wisdom measure emphasizing these aspects would, therefore, have a positive relationship with age (Glück, 2019a). In other words, if the different components of wisdom have different age trajectories, how a measure of wisdom is related to age would depend on which components of wisdom it emphasizes (Glück, 2019a; for an example, see Kunzmann et al., 2018).

To summarize, the integrative model can account for the situational variability of wisdom and for the differences between (more) personal and (more) general wisdom. In both cases, the key point is that the noncognitive components of wisdom influence the extent to which individuals can utilize wisdom-related knowledge, metacognitive capacities, and self-reflection in a given situation. The model also explains the low correlations between some wisdom measures and the different relationships of different wisdom measures with other variables. In addition, the model has implications for the design of wisdom-fostering interventions and for creating new, comprehensive measures of wisdom, as we discuss in the following.

### Implications for Designing Wisdom Interventions

Given the state of our world, it seems urgently important to devise effective ways to increase wisdom, both in our leaders and in the rest of us, on a short as well as on a long time scale (Ferrari & Kim, 2019; Glück, Sternberg, & Nusbaum, 2019; Sternberg & Hagen, 2019). The integrative model of wisdom has implications for the development of short-term and long-term interventions to foster wisdom. *Short-term interventions* increase a person’s wisdom in a given situation. They do not teach new knowledge; they activate knowledge and competencies that a person has but would not otherwise utilize. As mentioned earlier, experimental studies have demonstrated that people show higher levels of wisdom if they imagine talking to someone else about a problem (Staudinger & Baltes, 1996), imagine seeing a problem from a distance (Kross & Grossmann, 2012), or think about themselves in the third person (Grossmann & Kross, 2014). In the framework of the integrative wisdom model, these interventions activate the noncognitive wisdom components, especially open-mindedness.

Interestingly, a short-term intervention that did *not* have a general effect was reported by Glück and Baltes (2006). These authors made participants think about wisdom (e.g., by card-sorting characteristics of wise individuals) and then asked them to “try to give a wise response” to problems from the Berlin Wisdom Paradigm. At first sight, the intervention had no effect at all. An analysis of individual differences in outcomes, however, showed that some participants did profit from the intervention: those who were high in intelligence, life experience, and a personality factor labeled “self-regulation and openness toward growth.” Participants low in these variables actually responded *less* wisely than under standard conditions. From the perspective of the integrative wisdom model, these findings make sense: activating relatively abstract knowledge about wisdom does not necessarily translate into wise responses. Only individuals who have sufficient levels of both the cognitive and the noncognitive wisdom components will profit from such an intervention.

It would seem interesting to develop short-term interventions that focus directly on the noncognitive components of the integrative model, that is, an exploratory orientation, empathetic concern, or emotion regulation. Such interventions might, however, have stronger effects on wise behavior in real-life situations than on performance on theoretical problems as in the Berlin Wisdom Paradigm.

Long-term interventions, such as teaching for wisdom in schools (Sternberg, 2001), could foster both the noncognitive and the cognitive components of wisdom. For wisdom-related knowledge to be internalized, however, students probably need to be sufficiently emotionally engaged (Ardelt, 2004). As with most things we learn about life, explicit teaching may be less important than implicit teaching—“living wisdom” in the way we deal with our students (or our children). Introducing them to a variety of perspectives, cultures, and ways of living from early on, engaging them actively in finding solutions to difficult problems, and encouraging them to develop their own values and worldviews is likely to have stronger effects than making them listen to lectures about wisdom.

Based on findings on the situational variability of wisdom, the integrative wisdom model suggests a third way to increase wisdom in the world. Interventions targeting individuals are one important approach, but another possibility is to create situational contexts that enable or even enforce wise behavior (Glück, Sternberg, & Nusbaum, 2019). Both noncognitive components of wisdom, such as aiming for a common good, and cognitive components of wisdom, such as in-depth knowledge about a problem or active consideration of different viewpoints, can be “externally provided.” Democratic political systems, for example, are constructed to prevent one single individual or party from implementing unwise decisions. In addition to a carefully crafted balance of political institutions, independent institutions like a free press are important contributors to keeping dictatorial tendencies in check. As recent events in many democratic countries show, however, these balances are delicate. If the independence of such institutions is in jeopardy, democracies can start down a slippery slope toward dictatorship (Ambrose, 2019; Levitsky & Ziblatt, 2018). In our current era of political polarization, populist politicians can use the media to elicit strong emotions and in-group versus out-group thinking—which, according to our model, are antagonistic to wise reasoning and behavior (Glück, 2019b). In the face of these developments and an increasing need for global collaboration, we may need to rethink the robustness of our democratic institutions (Ambrose, 2019). On a smaller scale, to maximize the wisdom of decisions in businesses or institutions, it is important to create structures and cultures that ensure that many different voices are heard and taken seriously. Groups can be wiser than their members only if they (a) are heterogeneous in knowledge and perspectives *and* (b) value and utilize that heterogeneity (Surowiecki, 2005; for examples from the medical domain see Schwartz & Sharpe, 2019). Importantly, to be wise may mean to dissent rather than consent to a group consensus that does not adequately represent the complexity of a problem (see, e.g., Nemeth, 2018). Thus, the cognitive and noncognitive components of the model may need to work together not just to create individual wisdom, but also to enable wise thinking in groups.

### Implications for Measuring Wisdom

The fact that differences between measures can account for differences in empirical findings suggests that we have not yet managed to develop a comprehensive measure of wisdom that predicts wise behavior in real life. Wisdom is not exactly easy to measure (Glück, 2018). It varies by situation, which suggests multiple assessments rather than one-shot measures. It manifests itself in rare, difficult, emotionally challenging situations that cannot be emulated for measurement purposes. It includes cognitive and noncognitive components, which implicate different assessment methods. It includes self-reflection and humility, which may bias any kind of self-report. For all these reasons, the validity of existing measures of wisdom has been disputed (Ardelt, 2004; Brienza et al., 2018; Glück, 2018; Glück et al., 2013), and there is much room for innovative approaches. In the following, we discuss some implications of the integrative model for developing new measures of wisdom.

#### Outside versus inside perspectives

Some components of the integrative wisdom model can be measured from the outside, while others may only be accessible by introspection. It is not a good idea to ask participants to rate their own wisdom-related knowledge or metacognitive capacities; people are notoriously bad at judging their own competencies (Freund & Kasten, 2012), and the self-reflection paradox suggests that people who consider themselves very wise may just be very self-delusional (Aldwin, 2009; Glück, 2018). Measures like the Berlin Wisdom Paradigm or the Wise Reasoning Paradigm show that wisdom-related knowledge and metacognition can reliably be scored from interview transcripts or written responses. Whether a response balances different interests to maximize a common good should also be codable. Noncognitive aspects such as openness, empathic concern, or emotion regulation, however, may not necessarily be observable. Assessing all components of the integrative model may require a combination of self-report and open-ended measures. In addition to the content of problems or scale items, other aspects such as level of analysis (e.g., state vs. trait), informant (self vs. other), and domain (e.g., self, interpersonal, group, and general) may need to be considered to obtain more comprehensive wisdom measures.

#### Bringing wisdom measures closer to real life

One could argue that a measure of wisdom does not need to assess all components of the model. To measure wise behavior, it would be sufficient to put participants in a wisdom-requiring situation and see how they deal with it. This would, of course, be unethical as well as impractical. At the same time, the use of fictitious problems may elicit a person’s wisdom-related knowledge, but it may not tell us much about how well the person could utilize that knowledge in a real-life situation (Ardelt, 2004). As discussed earlier, researchers have suggested different routes toward more emotionally immersive measures, such as using videos of real-life conflicts (S. Thomas & Kunzmann, 2014), interviewing participants about difficult events from their past (Glück et al., 2019), having participants fill out a self-report scale with respect to a concrete past experience (Brienza et al., 2018), or having them imagine giving advice to a concrete other person (Hu et al., 2017). The central issue here is ecological validity, that is, the predictive power of wisdom measures for wise behavior in real-life situations. The integrative wisdom model can provide some guidance concerning the noncognitive components that ecologically valid measures should aim to capture. New technological possibilities may enable us to create higher levels of immersion in virtual problem simulations without putting too much emotional load on participants (Glück, 2018; Hu et al., 2017).

To summarize, we believe that the integrative wisdom model is a useful framework for the development of new interventions to foster wisdom and new measures to assess wisdom comprehensively in ecologically valid ways.

## Limitations of the Integrative Wisdom Model

The Integrative Wisdom Model is an attempt to integrate the thinking of many wisdom researchers into a comprehensive account of wise behavior. It is certainly not the end of the long-standing debate of how to best conceptualize wisdom, but we hope that it stimulates new research on wisdom in real life; in fact, we hope that the model will require further revision in a few years based on many new findings and insights. Despite its many promises, the model in its current form has several limitations, which we discuss in the following.

### Next Steps for Testing the Integrative Wisdom Model

While the Integrative Wisdom Model was built on a thorough review of the existing evidence, some of its parts have not yet been tested directly. In the following, we briefly discuss those parts of the model and possible studies to close the gaps.

#### Relationships between the trait components of the model

As Figure 1 shows, the model makes predictions about the relationships between the components of the model. In the language of structural equation modeling, it proposes two secondary factors, “non-cognitive wisdom” and “cognitive wisdom” that each comprise three factors. All components of the model have been operationalized as part of existing wisdom measures (see Tables 2 and 3) and could be directly assessed using those measures, except for the common-good orientation. This component, however, could both be coded from responses to wisdom problems quite easily and measured using value-orientation scales (see, e.g., Glück et al., 2020). Thus, a validation study could assess all trait components of the model and then test its proposed factor structure. Such a study would also provide new evidence on the relationship between the cognitive and noncognitive components at the trait level. Figure 1 suggests that they are independent for the sake of simplicity, and previous research has found low or zero correlations between cognitive-focused and noncognitive wisdom measures (e.g., Glück et al., 2013).

#### Conceptualizing and measuring wise behavior

To test the predictions of the model for wise behavior, we would first need a comprehensive characterization of wise behavior. Two lines of research may be particularly promising for developing a better understanding of wise behavior. First, we consider qualitative studies of actual real-life behavior to be very important. Such studies are high in ecological validity as they capture the complexity of real-life wisdom problems. A possible study design would be to compare high and low wisdom scorers on the ways they deal with real-life challenges or give advice to people faced with challenges. What questions do highly wise individuals ask that less wise individuals do not ask? Whom do they consult with for advice? How do they regulate their own and others’ emotions in a situation? How do they make decisions? How do they go about communicating their suggestions to the people involved? In addition to individual challenges, recent years have presented us with a host of collective challenges, such as climate change or the COVID-19 pandemic, which may provide interesting “laboratories” for studying wise behavior and advice-giving. One advantage of such research is that it may also provide us with wise ideas about how to deal with the respective challenges.

The second line of research, which was already mentioned in the section on new wisdom measures, would utilize new technologies to design more complex experimental situations, for example, in virtual reality (Hu et al., 2017). Studies presenting problems in virtual environments, where participants can collect information, make decisions, and solve problems, may enable us to study wise “behavior” in more controlled but somewhat less realistic and immersive settings.

Once reliable and valid indicators of wise behavior have been established, the propositions of the model concerning the prediction of wise behavior can be tested more directly. A particularly interesting question would be whether the cognitive and noncognitive components influence wise behavior only through wise reasoning, which would mean that all wise behavior is reasoning-based, or whether there is an additional direct pathway from the non-cognitive components to wise behavior. Another interesting question is whether the relative importance of the trait components of the model varies according to the characteristics of the respective problem. In sum, while the new model integrates the corpus of existing evidence, future research will certainly provide new insights that will lead to modifications and extensions of the model.

### What the Model Does Not Cover (for Now)?

The model describes wise behavior in one particular class of situations, namely, difficult, uncertain, emotionally challenging real-life situations. Studies on people’s experiences with real-life wisdom suggest that this type of situation may be where wisdom manifests itself most clearly, as the demands exceed most people’s regulatory capacities (Glück et al., 2005; Montgomery et al., 2002; Yang, 2008). However, wisdom also affects many other aspects of a person’s life. According to both modern and ancient philosophers, wisdom is about knowing how to live a good life, and a good life, fortunately, does not only consist of dealing with difficult problems. Studies suggest that wise individuals feel that they are living the life that is right for them (Weststrate & Glück, 2017b), that they are more grateful for the good things in their life than other people (König & Glück, 2014), and that they are both high in eudaimonic well-being and satisfied with their lives (Ardelt, 2019; Ardelt & Edwards, 2016; Ardelt & Jeste, 2018; Bauer et al., 2019; Glück et al., in press). Outside the challenging situations that this article looks at, wise people probably make many small decisions every day that are guided by self-directional, benevolent, and universalistic values (Glück et al., 2020) and thus contribute to their own well-being and that of others. At the same time, as Weststrate and Glück (2017b) have argued, there is a certain tension between the ideas that wisdom increases well-being and that wisdom involves dealing with the darker sides of human existence. In fact, caring about a common good may lead wiser individuals to be more, not less, emotionally affected by the suffering of others, leading them to act and intervene in situations that others may choose to ignore.

The Integrative Wisdom Model does not cover these aspects of wisdom. It focuses on wisdom in challenging situations because it seems particularly important to investigate how wise behavior can be fostered in those situations where it is most needed and at the same time least likely to occur. We hope that it is a first building block of a broader understanding of how wisdom manifests itself in people’s lives.

### Complexity and (at the Same Time) Simplicity of the Model

Figure 2 displays the Integrative Wisdom Model in a decision-tree format, illustrating that it includes two moderators: (a) the noncognitive wisdom components moderate which state of mind a wisdom-requiring problem elicits in a person, and (b) that state of mind moderates the extent to which the person is able to utilize their cognitive wisdom resources in the situation. This double moderator effect renders the model quite complex. To some extent, this complexity is simply due to the fact that wisdom, as it turns out, is highly complex—maybe wise behavior in real life is rare because it requires a complex combination of qualities and conditions. It may be close to impossible to test all parts of the model in one integrative study, but new empirical evidence may close those gaps that are not yet well-studied.

Despite the complexity of the model, some aspects of it are certainly simplified in comparison to reality. As mentioned earlier, Figure 1 displays the cognitive and noncognitive components as independent. In reality, however, the components are probably interrelated. According to developmental models of wisdom, noncognitive qualities such as openness or empathy foster the development of wisdom because they motivate individuals to reflect on experiences, thus leading to the accumulation of wisdom-related knowledge, which, in turn, fosters people’s self-reflective and self-regulatory capacities (Baltes & Staudinger, 2000; Glück & Bluck, 2013; Glück et al., 2019). Thus, the noncognitive and cognitive components of wisdom may not only interact in producing wise behavior in difficult real-life situations, but also co-develop, strengthening one another over time, becoming a “self-reinforcing syndrome” (Glück et al., 2019, 364).

Because the interplay of the cognitive and noncognitive components of the model is more bidirectional than it may look in the model, the unidirectional path from the wise state of mind to the utilization of cognitive wisdom resources is probably too simple as well. For example, Brienza et al. (2021) showed that wise reasoning fostered more positive attitudes toward outgroups and less polarization of attitudes across different settings and study designs. It seems very plausible that the (cognitive) ability to take a step back and consider different perspectives in a situation may also lead to lower emotional arousal. Similarly, even recognizing the need for acting wisely in a seemingly clear-cut situation may already require components of wisdom such as awareness of uncertainty and reflectivity. Our model, complex as it is, probably simplifies the actual “orchestration of mind and virtue” (Baltes & Staudinger, 2000, p. 129) that wisdom involves.

In fact, some of the model components were difficult to classify as either cognitive or noncognitive. For example, consistent with the findings of Brienza et al. (2021), emotion regulation certainly has a cognitive component; our narrative research shows wise people’s capacity to consciously reflect on their feelings in a situation and on strategies for dealing with them. Such strategies could also be classified as self-reflection. We decided to classify the resources essentially based on whether they contribute to a person’s emotional and motivational state in the situation or to the cognitive capacities they use in dealing with the problem. For this reason, self-reflection was classified as cognitive, although it has a strong motivational component.

### Does Wisdom Even Exist in the Real World?

Psychological accounts of wisdom have a tendency to sound lofty and unrealistic—it seems like ideally wise individuals never get angry or depressed, care deeply even about their enemies (if they have any enemies at all) and are able to find perfect solutions to problems of infinite complexity (see Ardelt et al., 2019; Baltes & Kunzmann, 2004). People’s beliefs about wisdom exemplars such as Solomon or Gandhi may have little to do with who those individuals actually were (Grossmann & Kross, 2014). Such ideals are unlikely to be attainable by any human being. The word “wise” may indeed be a label that people tend to reserve for extraordinary individuals. We believe that one of the functions of models like the current one is to explain how rare behavior can arise from a constellation of cognitive and noncognitive qualities that are each continuous and that can co-develop into a broader quality that is more than the sum of its parts.

The rarity of high levels of wisdom is, of course, also a challenge to empirical research. We typically do not find many highly wise participants in representative studies. However, nomination approaches may enable researchers to put together samples in which wisdom is not ubiquitous but at least overrepresented (see Baltes et al., 1995; Glück et al., 2013, 2019). We consider it as highly important, however, not to equate wisdom research with studying a very small group of very special individuals. The facts that wisdom varies across situations (e.g., Grossmann, 2017) and is accumulated over time (e.g., Glück et al., 2019) suggest that it may be much more important to study how we can foster wisdom both by creating wisdom-supportive contexts and by developing effective long-and short-term wisdom interventions.

### The Need to Consider Cultural Aspects of Wisdom

Another important limitation of the integrative wisdom model concerns the question of its cultural generalizability. The model is based on wisdom conceptions developed by “Western” researchers and findings from studies in “Western,” relatively individualistic societies. While we believe that the components of the model are also part of “Eastern” conceptions of wisdom (see, e.g., Ferrari & Alhosseini, 2019; Yang & Intezari, 2019), relative emphases may differ and/or some components may be missing from the model. One could argue that cultures as a whole differ in their levels of the different components of wisdom (e.g., Asadi et al., 2019; Atwijukire & Glück, 2020; Grossmann et al., 2012)—for example, that people in collectivistic cultures are, on average, higher on concern for others and lower on self-knowledge. While people from highly individualistic cultures become less self-focused as they develop wisdom, maybe people from highly collectivistic cultures become more aware of their personal strengths and needs. In that sense, wisdom might represent a largely culture-independent ideal of how human beings live a good life that manifests differently depending on the biological, environmental, social, and cultural conditions into which people are born.

## The Relationship of the Integrative Wisdom Model to Other Overarching Models of Wisdom

This article is not the only one that has recently aimed to review and integrate the various conceptions of wisdom and integrate existing models. Grossmann, Weststrate, Ardelt, et al. (2020) proposed a common-denominator model that includes only those components of wisdom that showed the highest degree of agreement in a survey of wisdom researchers. Thus, while the integrative model aims to systematically consolidate different wisdom components from many different wisdom theories, the common-denominator model focuses on those components that are robustly represented across theories. The common core of wisdom, according to that model, is the use of metacognition for morally grounded reasoning about complex problems. Sternberg and Karami (2021) proposed a so-called “6P” model of wisdom, drawing on Rhodes’s (1961) classical distinctions of four perspectives on creativity. Rather than proposing an overarching model, they suggested classifying existing wisdom models according to whether they look at the (a) *Purpose* of wisdom, (b) the environmental/situational *Presses* that foster wisdom, (c) the kinds of *Problems* that require wisdom, (d) the characteristics of *P*ersons who are wise, (e) the psychological *Processes* that contribute to wisdom, and (f) the *Products* of wisdom. The main aim of the 6P model is not to explain wisdom or wise behavior, but to provide a framework to structure existing wisdom research and identify important gaps in our knowledge. Finally, Karami et al. (2020) proposed the polyhedron model of wisdom based on a systematic review of existing wisdom research. They argued that wisdom is a situational construct that involves the adequate use of knowledge, intelligence and creativity, self-regulation, openness and tolerance, altruism and moral maturity, and sound judgment to solve critical problems. The components of the polyhedron model are largely consistent with those of the Integrative Wisdom Model, except for the additional component of creativity. The Integrative Wisdom Model goes beyond the polyhedron model in that it specifies how the noncognitive and cognitive components of wisdom interact in creating wise behavior, thus allowing for a better understanding of some of the underlying mechanisms and more specific hypotheses for empirical research.

## Conclusion

The Integrative Wisdom Model presented here is an attempt, based on the current state of research, to show how different psychological conceptions can be integrated into a framework that explains wise behavior. Our main goal was to show that the different models of wisdom proposed by researchers complement rather than contradict one another and that combining their different elements may enable us to explain wise behavior in real life. Many questions remain open, and if, as we very much hope, wisdom research continues to attract new researchers bringing new backgrounds and perspectives into the field, the model will certainly have to be thoroughly revised in five to ten years. If we want to understand a phenomenon as complex and multifaceted as wisdom, we should heed Surowiecki’s (2005) recommendations for maximizing group wisdom: bring together as heterogeneous a group of people—in this case, researchers—as possible, and listen to all their voices. We hope that this article is a step in this direction.

## Notes

1. We thank an anonymous reviewer for bringing up this question and for providing the helpful example of aggressive behavior.
